# Discovery of therapeutic targets for spinal cord injury based on molecular mechanisms of axon regeneration after conditioning lesion

**DOI:** 10.1186/s12967-023-04375-1

**Published:** 2023-07-28

**Authors:** Xiaoxiong Wang, Wenxiang Li, Jianping Zhang, Jinze Li, Xianjin Zhang, Min Wang, Zhijian Wei, Shiqing Feng

**Affiliations:** 1 Department of Orthopedics, Qilu Hospital of Shandong University, Jinan, Shandong China; 2 Shandong University Centre for Orthopaedics, Advanced Medical Research Institute, Cheeloo College of Medicine, Shandong University, Jinan, 250012 Shandong People’s Republic of China; 3 International Science and Technology Cooperation Base of Spinal Cord Injury, Tianjin Key Laboratory of Spine and Spinal Cord, Department of Orthopedics, Tianjin Medical University General Hospital, Tianjin, People’s Republic of China; 4 Tianjin Key Laboratory of Lung Cancer Metastasis and Tumor Microenvironment, Tianjin Lung Cancer Institute, Tianjin Medical University General Hospital, Tianjin, 300052 People’s Republic of China; 5 University of Health and Rehabilitation Sciences, No.17, Shandong Road, Shinan District, Qingdao, 266071 Shandong People’s Republic of China; 6 Department of Orthopedics, Tianjin Medical University General Hospital, No154. Anshan Rd, He Ping Dist, Tianjin, 300052 China

**Keywords:** Dorsal root ganglia, Axonal regeneration, Conditioning lesion, Spinal cord injury, Telmisartan, Bioinformatical analysis, Differentially expressed genes

## Abstract

**Background:**

Preinjury of peripheral nerves triggers dorsal root ganglia (DRG) axon regeneration, a biological change that is more pronounced in young mice than in old mice, but the complex mechanism has not been clearly explained. Here, we aim to gain insight into the mechanisms of axon regeneration after conditioning lesion in different age groups of mice, thereby providing effective therapeutic targets for central nervous system (CNS) injury.

**Methods:**

The microarray GSE58982 and GSE96051 were downloaded and analyzed to identify differentially expressed genes (DEGs). The protein–protein interaction (PPI) network, the miRNA-TF-target gene network, and the drug-hub gene network of conditioning lesion were constructed. The L4 and L5 DRGs, which were previously axotomized by the sciatic nerve conditioning lesions, were harvested for qRT-PCR. Furthermore, histological and behavioral tests were performed to assess the therapeutic effects of the candidate drug telmisartan in spinal cord injury (SCI).

**Results:**

A total of 693 and 885 DEGs were screened in the old and young mice, respectively. Functional enrichment indicates that shared DEGs are involved in the inflammatory response, innate immune response, and ion transport. QRT-PCR results showed that in DRGs with preinjury of peripheral nerve, Timp1, P2ry6, Nckap1l, Csf1, Ccl9, Anxa1, and C3 were upregulated, while Agtr1a was downregulated. Based on the bioinformatics analysis of DRG after conditioning lesion, Agtr1a was selected as a potential therapeutic target for the SCI treatment. In vivo experiments showed that telmisartan promoted axonal regeneration after SCI by downregulating AGTR1 expression.

**Conclusion:**

This study provides a comprehensive map of transcriptional changes that discriminate between young and old DRGs in response to injury. The hub genes and their related drugs that may affect the axonal regeneration program after conditioning lesion were identified. These findings revealed the speculative pathogenic mechanism involved in conditioning-dependent regenerative growth and may have translational significance for the development of CNS injury treatment in the future.

**Supplementary Information:**

The online version contains supplementary material available at 10.1186/s12967-023-04375-1.

## Background

Axons in the mature mammalian central nervous system do not regenerate after injury, while axons in peripheral nerves show a remarkable regenerative ability. The regeneration capacity of severed axons is directly related to the restoration of function. The failure of axons to regenerate after CNS injury usually leads to permanent loss of motor and sensory nerve functions [1, 2]. To this end, exploring the mechanisms of peripheral nerve axonal regeneration may be a breakthrough point in the difficulty of repairing the CNS after injury.

The regenerative capacity of neuronal axons is not static, which is fully demonstrated by axonal regeneration of the dorsal root ganglion (DRG) neuron after sciatic nerve injury (SNI). DRG sensory neurons are pseudo-unipolar neurons with two branches. When DRG axonal branches are damaged, the peripheral axonal branch regenerates into the peripheral nerves, but the central axon branch only regenerates to the dorsal root entry zone-spinal cord junction and fails to enter the dorsal column of the spinal cord unless the peripheral branch is primed [3, 4]. After dorsal column lesions, all fibers stop at the spinal cord injury site. However, as an intermediate station connecting the spinal cord and peripheral nerves, the unique structure of the dorsal root ganglion allows the regeneration of the axons after SCI [5]. What is of particular interest is that if the peripheral branches of DRG are first lesioned, followed by cutting the dorsal column axons 1 week later, these central axons of DRG can now regenerate into and beyond the lesion site following adult spinal cord injury. Injury to the peripheral branches of the DRG activates the axonal regeneration program in the central branches, whereby pre-injury to the peripheral branches is termed a conditioning lesion of the DRG [6]. Molecular changes in these DRG neurons are induced early in the conditioning lesion, allowing their central axonal branches to grow in a typically hostile and inhibitory environment [7, 8]. Previous studies have induced axonal regeneration in the spinal cord by mimicking the conditioning effect of peripheral injury [3, 9–11]. This “conditioning lesion effect” makes the dorsal root ganglion an ideal model for studying the mechanism that modulates the neuronal regeneration program after axotomy, providing a new direction for SCI repair. However, the underlying mechanism that triggers axon regeneration after the effect of conditioning lesion remains unclear.

The capacity to regenerate damaged axons is not only related to the identity of neurons but also influenced by their age. With the increase of age, neurons in the central nervous system of mammals lose their inherent ability to regenerate [2, 12, 13]. Moreover, there is also a similar decline in age-associated peripheral nerve regeneration. Compared with the young animals, the axon regeneration rate and density decreased in the aged animals after peripheral nerve injury [14, 15]. The axons of young sensory dorsal root ganglion neurons have been shown to own a higher intrinsic regeneration capacity than aging axons [16]. Comparative analysis of embryonic and adult DRG axon microarrays reveals embryonic DRG axon mRNAs are enriched in encoding cytoskeletal-related proteins with a role in axonal outgrowth [17]. In contrast, adult DRG axons are enriched in transcripts encoding immune molecules with a function in nociception [18]. However, the entire repertoire of transcripts present in different age DRG after SNI and how this pool of mRNAs changes dynamically during aging still need to be explored.

**Fig. 1:**
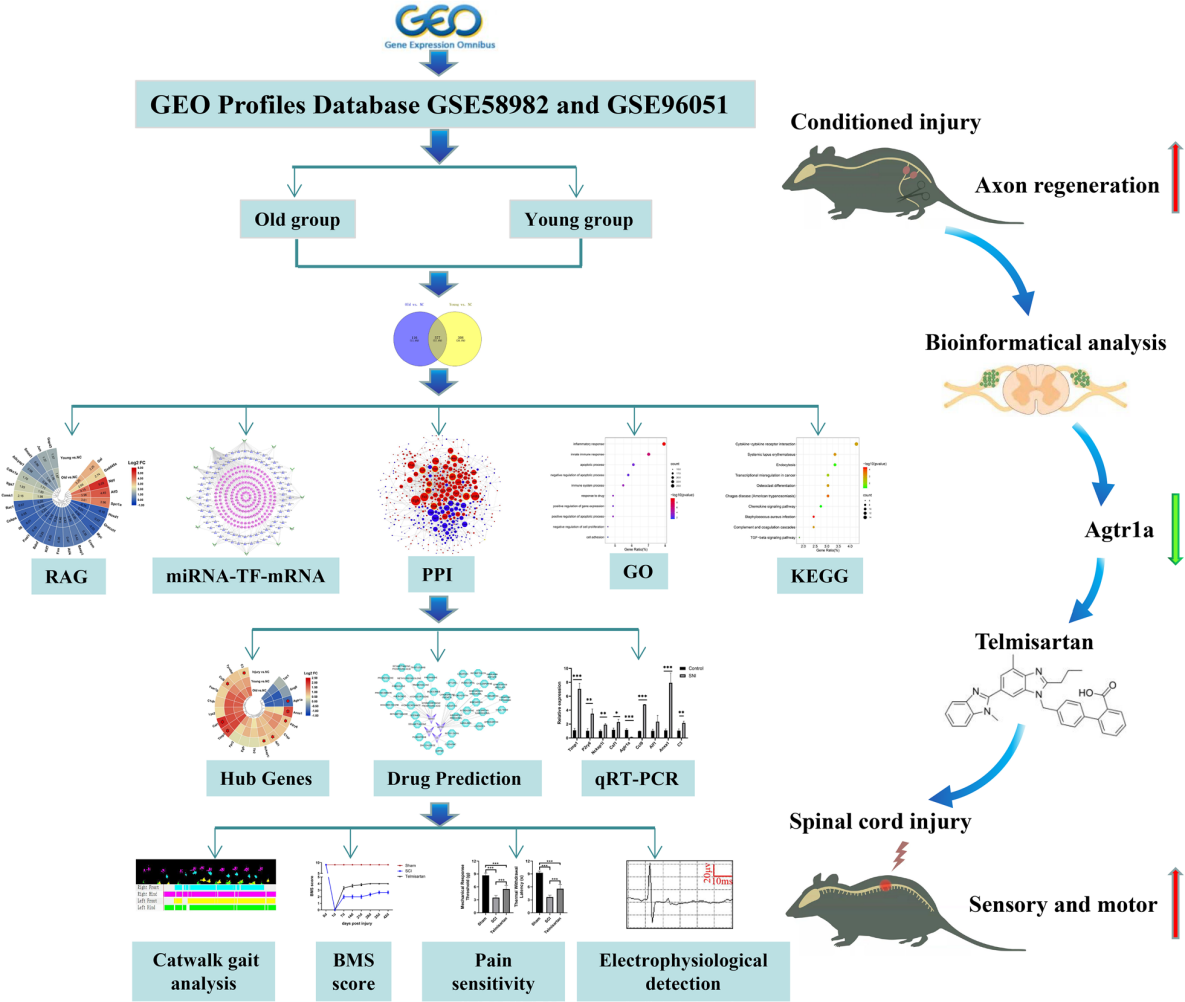
The process flow diagram of this study

Regeneration of DRG sensory afferent axons is indispensable for sensory and motor recovery after SCI, and the regenerative potential of these axons generated by the conditioning lesion is a key step towards improved functional recovery. Given this, our team explored the effective therapeutic targets for SCI by starting with the effect mechanism of conditioning lesion. In recent years, microarray and RNA sequencing (RNA-seq) techniques have been widely used in transcriptome analysis to study the biological mechanism of diseases. In the current study, we systematically downloaded and analyzed the GSE58982 and GSE96051 datasets from the gene expression comprehensive (GEO) database by the bioinformatics method. The present study not only provides a comprehensive map of transcriptional changes following conditioning lesion but also elucidates how it differs in young and old DRGs of mice. We pioneered the construction of the drug-hub gene network and identified that the expression of Agtr1a is downregulated after conditioned DRGs, which may be a potential target of axon regeneration. Then, we verified its expression in the DRGs and predicted the related-drug telmisartan. Due to the effect of telmisartan, an angiotensin II type 1 receptor antagonists, can suppress Agtr1a expression [19, 20], which mimics the key step of DRGs response to SNI. Further, in vivo experiments showed the effectiveness of telmisartan on the recovery of sensory and motor functions after SCI **(**Fig. 1**)**. Overall, we shed light on the intrinsic molecular mechanisms to reprogram DRG neurons into the regenerative program following the effects of conditioning lesion, thereby providing new insights into the treatment of CNS injury.

## Methods

### Animals

A total of 48 adult C57BL/6 mice weighing 20 ± 3 g (8-week-old) were purchased from the Radiation Study Institute‐Animal Center (Tianjin, China). All animals were maintained on a 12/12 h light/dark cycle under a temperature of 20–25 °C and humidity around 50%. This study and all experimental procedures were approved by the Ethics Committee of the Institute of Radiation Medicine, Chinese Academy of Medical Sciences (Approval number: IRM-DWLI-2019110) and performed according to the national guidelines for laboratory animal use and care.

### Acquisition of GEO datasets

The microarray dataset GSE58982 and GSE96051 were retrieved from GEO (https://www.ncbi.nlm.nih.gov/geo/). As an experimental microarray dataset, GSE58982 was submitted in 2014 by Michio Painter et al. [21]. Its platform is GPL6885 (Illumina Mouseref-8 V2.0 expression Beadchip). The GSE58982 dataset contained 11 samples (young, 2-month-old mice, n = 3 in the surgery group vs n = 3 in the control group; old, 24-month-old mice, n = 3 in the surgery group vs n = 2 in the control group). Total RNA was taken from L4 and L5 dorsal root ganglia from old and young mice at 5 days after sciatic nerve crush injury. The microarray dataset GSE96051, as a validated dataset, was deposited by Kevin Huang et al. [22]. and contained 9 samples (adult mice, n = 5 in the surgery group vs n = 4 in the control group). Total RNA was taken from L4 DRG at 3 days after sciatic nerve transection. The RNA was measured by microarray (Platform: GPL7202 Agilent-014868 Whole Mouse Genome Microarray 4 × 44 K G4122F).

### Screening of DEGs and functional enrichment analysis

We downloaded a series of matrix files and platforms of GSE58982 and GSE96051 datasets in TXT format from the GEO database. Based on the R language software (v.3.5.3: https://www.r-project.org), all extracted files were processed, and all probe IDs were converted into gene symbols. Probes without matching gene symbols were excluded from the study. If a gene symbol had multiple probes, we analyzed the average expression levels of these gene symbols. The quality of gene expression data was analyzed and visualized for each sample set using the ggplot2 R package. To identify DEGs in GSE58982 and GSE96051 datasets between the surgery group and control group in the different age groups, we utilized the limma R package in Bioconductor [23].Genes with |logFC|> 0.5 and adjusted P-value < 0.05 were considered significant. To filter shared and unique DEGs between the different groups, we submitted the list of DEGs to Venny2.1 (http://bioinfogp.cnb.csic.es/tools/venny/index.html). Then, we used the online bioinformatics resource DAVID v6.8 (https://david.ncifcrf.gov) to perform GO and KEGG analysis on different groups of DEGs at the functional level [24].

### Construction of the miRNA-TF-target gene network

TRRUST V.2 (https://www.grnpedia.org/trrust/) is an open, manually curated database of human and mouse transcriptional regulatory networks [25]. We downloaded the mouse transcription factor (TF) and targeted gene interaction pairs and intersected these TFs with shared DEGs. Then, the overlapped TFs verified by GSE96051 datasets were used to construct the miRNA-TF- target gene network.

ENCORI (https://starbase.sysu.edu.cn/index.php) has identified more than 2.5 million miRNA-mRNA interactions from multidimensional sequencing data [26]. We retrieved and downloaded miRNA-mRNA interactions from ENCORI and then screened out miRNAs that interacted with the TF genes obtained above.

### Construction of the PPI networks and screening hub genes

To fully understand the regulation mechanism of dorsal root ganglion (DRG) neuron regeneration triggered by SNI, it is necessary to study its constituent proteins and their functional interactions. The STRING V.11 (http://string-db.org/) database aims to collect, score, integrate and supplement publicly available information on protein–protein interactions through computational predictions, and to establish a comprehensive network of physical and functional interactions [27]. We upload the list of differential genes to the Multiple Protein web of the STRING database and set the interaction score > 0.4 as the threshold to obtain the TSV file of string interactions. Cytoscape is an open-source visualization software, the core of which is to visually layout and integrate the network and expression profile [28]. The plugin cytoHubba of Cytoscape can measure nodes by their network characteristics to infer the core elements of biological networks. The DEGs whose degree score was ≥ 30 were set as hub genes. To add credibility, hub genes validated in the GSE96051 dataset were selected for further analysis.

### DGIdb drug prediction

The Drug Gene Interaction Database (DGIdb) (http://dgidb.genome.wustl.edu/) is designed to help researchers predict targeted genes or gene products that interact with drugs, which ideally has therapeutic benefits for patients [29]. The hub genes selected as potential targets for medicinal drugs were uploaded to the DGIdb online tool. Filters were set to include “FDA Approved”, 22 databases, 43 gene categories, and 31 gene interaction types.

### Sciatic nerve axotomy

Mice were randomly assigned to surgery groups and sham groups (n = 6 in each group). All sciatic nerve axotomy procedures were standardized and performed under aseptic conditions. Mice were anesthetized with 4% isoflurane until unconscious followed by 2% isoflurane during surgery. For the surgery group, the mice were made a small skin incision in the middle of the thigh, and the soft tissue was separated by blunt dissection. Then, the sciatic nerve was exposed and transected. For the sham group, the mice were only made a small skin incision by surgical scissors without sciatic nerve axotomy. The mice were deeply anesthetized to harvest DRGs (L4 and L5) at 5 days post-operation.

### SCI model and telmisartan treatment

Mice were randomly assigned to three groups (n = 6 in each group): Sham group, laminectomy and 0.9% NaCl; SCI group, spinal cord contusion and 0.9% NaCl; Telmisartan group, spinal cord contusion and telmisartan (HY-13955, MCE, Shanghai, China). The spinal cord contusion model was established with the MASCIS Impactor Model III (The State University of New Jersey, USA). Briefly, the mice were weighed, then anesthetized with isoflurane by inhalation anesthesia. The spinous process of thoracic vertebra 8 (T8) was located by the anatomical landmarks of the mouse body surface, and the spinal cord was exposed after laminectomy. Subsequently, the spinal cord was impacted by the MASCIS Impactor Model III with a height of 12.5 mm. The incision was sutured and sterilized with an iodophor cotton ball. In comparison, the sham operation group had undergone only laminectomy with no spinal cord contusion. The oral solution of 0.9% NaCl and telmisartan (5 mg/kg/day) were administered to different groups of mice by oral gavage for 2 consecutive weeks after 1 day of SCI every morning (10 am).

### Assessment of locomotor function and pain sensitivity

Basso Mouse Scale (BMS): To evaluate the recovery of hindlimb locomotor behavior in mice, BMS scores were performed before SCI, on days 1, 7, and once per week thereafter for 6 weeks post SCI according to guidelines of the Basso Mouse Scale [30]. The experiments were carried out by two observers blinded to the experimental groups and the assessments were repeated three times and recorded immediately. Mice from different experimental groups were randomly tested and scored. The mouse was individually placed in the open field and observed by two investigators for 4 min. The scores of the BMS ranged from 0 to 9, with each score representing a different degree of hindlimb locomotory behavior. The scores were based on multiple parameters of hindlimb movements such as joint movements, paw position, stepping pattern, coordination, trunk stability, and tail control.

### Assessment of mechanical and thermal sensitivity test

Measurement of mechanical allodynia and thermal sensitivity of mice were carried out at 6 weeks after SCI as the methods described previously [31]. Mechanical sensitivity test: The hind paw withdrawal threshold to acupuncture pain stimulation was determined using von Frey filaments. Mice were placed on an elevated wire mesh screen and restrained with a transparent glass compartment to allow the researchers to observe and free access to the plantar surface of the paws. When the mouse was not paying attention to the tester or stimulus, the left and right hind paws were tested in random order using the up-down method [32]. 15 consecutive trials were performed on each hind paw. The 50% withdrawal threshold was defined as the lowest stimulus that caused withdrawal in at least half of the trials. Since this test stimulated the same area of skin as the plantar heat test, the two tests were performed on different days. Thermal sensitivity test: Mice were placed in individual transparent plastic compartments. When the mouse was not paying attention to the tester or stimulus, a 25 watts infrared radiant heat source was applied through a glass floor to the middle of the plantar surface of the hind paw. When the mouse lifted its paw, the heat source stopped automatically and withdrawal latency was recorded. Five trials were performed on each of the left and right hind paws, with at least one minute interval between each trial. The mice were observed for behavior during stimulation, including sniffing, licking, looking at the affected paw, or attacking the stimulus. All behavioral tests were blindly performed for the entire experiment.

### Catwalk automated quantitative gait analysis

The footprints and locomotor gait dynamics of the mice were assessed by the CatWalk XT system (Noldus Information Technology, the Netherlands) 6 weeks after SCI as the methods described previously [33]. The CatWalk XT system consists of a walking platform with a transparent glass plate illuminated by a green LED light and a high-speed camera capturing the real footprints. At the top of the walking platform is a ceiling with a red LED background, which visualizes the body contour of the mouse. The CatWalk testing was performed in a dark, quiet environment. The animals were trained on the device at least twice prior to surgery. Before the formal testing, we trained the animals every other day in order to adapt to the environment. During the formal testing, the motor activity of the mice was recorded at least three times, provided that the calibration parameters were the same for each group. We defined a duration greater than 5 s but less than 20 s and a speed change ≤ 60% as a compliant run. The footprints of each mouse were recorded by the digital video camera blow the glass and were analyzed by the CatWalk XT software. Using this system, a series of parameters was automatically analyzed, including stride length, stands, max contact area, and base of support.

### Electrophysiology

To assess the nerve function recovery of mice after SCI, electrophysiological detection (YRKJ-G2008; Zhuhai Yiruikeji Co, Ltd, Guangdong, China) was applied to examine motor evoked potential (MEP) at 6 weeks after SCI. Mice were anesthetized at 6 weeks after injury as above described. The stimulating and reference electrodes were put under the skin between the ears, the ground electrode under the skin of the center of the back, and the recording electrode percutaneously in the gastrocnemius muscle of the lower limbs to record peak amplitude and latency. A 5-mA stimulation was administered to stimulate the motor region of the cerebral cortex. The amplification gain was 2000. MEPs were evoked approximately every 15 s by transcranial stimulation. Peak-to-peak amplitudes of MEP from 3 stimulations were recorded to assess intra-animal variability and reproducibility.

### qRT-PCR validation

Validation of the hub genes by qRT-PCR was performed on DRG 5 days after SNI. Total RNA extraction from pre-conditioned and sham DRGs samples with a TRI® Reagent (Solarbio, China). Complementary DNA (cDNA) was transcribed reversely from total RNA using a Prime Script RT Master Mix (Takara, Japan). The mRNA level was assessed using SYBR‐Green Premix Ex Taq (Takara) following the protocol. QRT‐PCR was carried out on LightCycler® 96 System (Roche, China), and the relative expression of the hub genes was calculated by the 2 − ΔΔCt method with the GAPDH acting as the internal control. The sequences of primers are available in Table 1.

**Table 1:** Primer sequences

Gene	Forward primer sequence (5′-3′)	Reward primer sequence (5′-3′)
Aif1	ATCAACAAGCAATTCCTCGATGA	CAGCATTCGCTTCAAGGACATA
C3	CCAGCTCCCCATTAGCTCTG	GCACTTGCCTCTTTAGGAAGTC
Nckap1l	TGTCCGAAATAGCACGCAACA	ATCCCGAAATTCCATGACATCC
P2ry6	GTGAGGATTTCAAGCGACTGC	TCCCCTCTGGCGTAGTTATAGA
Ccl9	CCCTCTCCTTCCTCATTCTTACA	AGTCTTGAAAGCCCATGTGAAA
Timp1	GCAACTCGGACCTGGTCATAA	CGGCCCGTGATGAGAAACT
Anxa1	ATGTATCCTCGGATGTTGCTGC	TGAGCATTGGTCCTCTTGGTA
Agtr1a	AACAGCTTGGTGGTGATCGTC	CATAGCGGTATAGACAGCCCA
Csf1	ATGAGCAGGAGTATTGCCAAGG	TCCATTCCCAATCATGTGGCTA
GAPDH	AGGTCGGTGTGAACGGATTTG	TGTAGACCATGTAGTTGAGGTCA

### Immunofluorescence staining

Spinal cord tissues were harvested, fixed, and dehydrated at 6 weeks after SCI. The dehydrated spinal cords were embedded in OCT, followed by sectioning into 6 µm slices (Leica, CM3050S). The sections were blocked and permeabilized with 5% goat serum and 0.25% Triton X-100 in PBS for 1 h at RT. The sections were incubated with primary antibody overnight at 4 ℃. Primary antibodies used were rabbit anti-neurofilament heavy polypeptide (NF200, 1:2000, Abcam, ab8135) and anti-serotonin (5-HT, 1:5000, Immunostar, 20080). The next day, the sections were incubated with secondary antibodies Alexa Fluor 488 or 555-labeled goat anti-rabbit IgG (H + L) (1:500, Invitrogen) for 1 h at RT. Nuclei were labeled using DAPI (Abcam, ab104139). The sections were examined and photographed using fluorescence microscopy (Olympus, VS120). Quantitative analyses of relative fluorescence intensities were performed using Image J software.

### Western blot

At 5 days post-SCI, 15 mg of spinal cord tissue was obtained from the injury center and fully lysed in a RIPA lysate (Beyotime Biotechnology, P0013B) containing a protease inhibitor (Roche, 04693132001). Protein concentration was determined using a BCA kit (Biosharp, BL52A). The denatured protein samples were isolated using SDS-PAGE (Epizyme Biotechnology, PG113). Subsequently, the protein samples were transferred to a PVDF membrane (Millipore, ISEQ00010). After blocking the membrane with 5% skim milk (Beyotime Biotechnology, P0216) for 1 h at room temperature, the bands were incubated with primary antibody at 4 ℃ overnight. Primary antibodies used were mouse anti-angiotensin II type 1 receptor (AGTR1, 1:1000, Abmart, M023088S) and anti-β-Actin (1:5000, Cell Signaling Technology, #3700S). The next day, after the bands were washed using TBST, they were incubated with peroxidase-conjugated rabbit anti-mouse secondary antibodies. Enhanced chemiluminescence (ECL, Millipore, Billerica, MA, USA, Cat# WBKLS0500) was used to view blots. Protein quantification and analysis were performed using the Image J software.

### Statistical analysis

Statistical analyses were performed using GraphPad Prism 8.0 software (San Diego, CA, USA). Comparisons between two groups were analyzed using the unpaired t-test. One-way or two-way analysis of variance with Tukey’s post hoc test was used to analyze the differences among multiple groups or between the groups over time. Data were represented by the mean ± SEM. A value of P < 0.05 was considered statistically significant.

## Results

### Peripheral injury induces transcriptional responses in the old and young DRGs

**Fig. 2:**
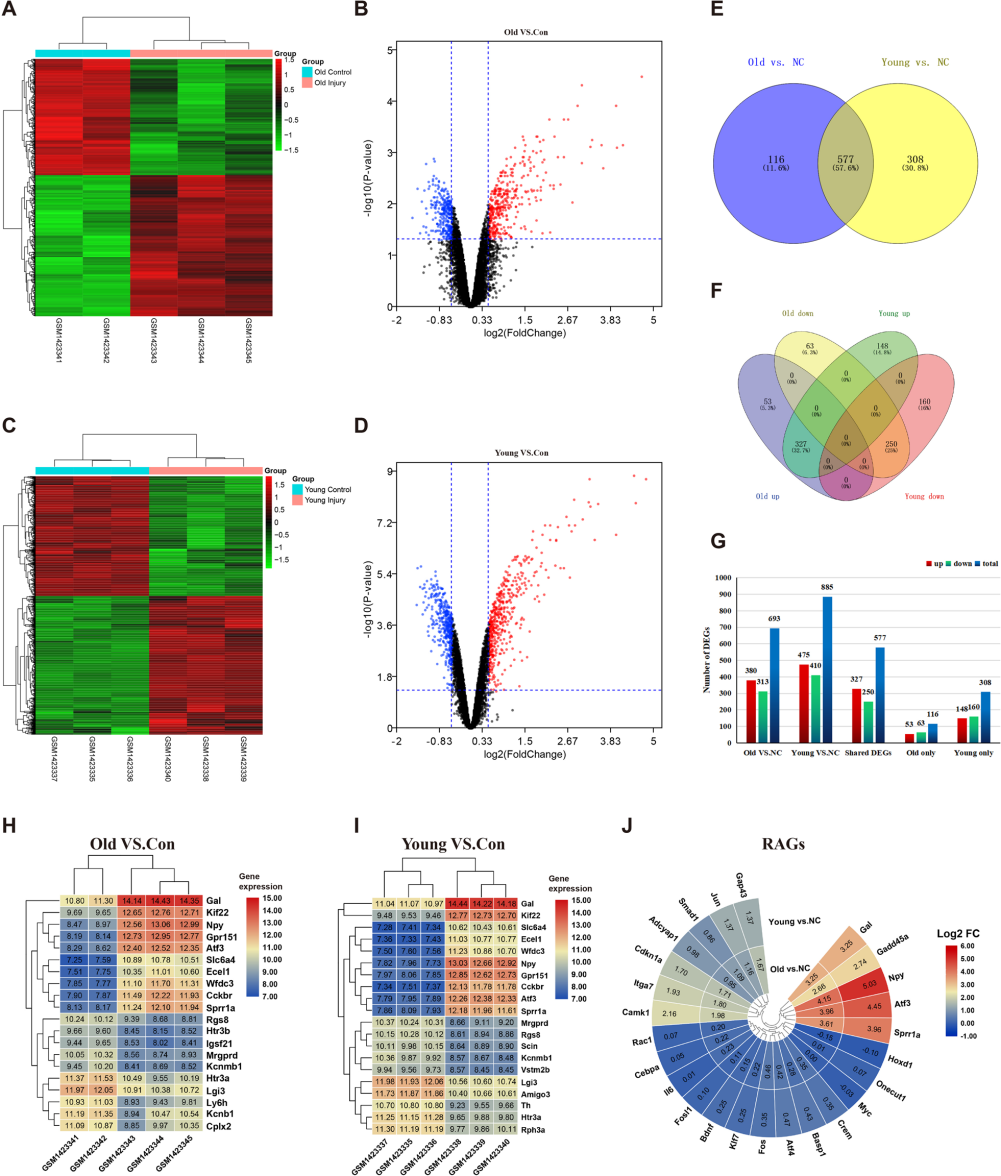
Characterization of DEGs between young/old surgery and normal control DRG samples. **A** Heatmap of DEGs in the old group; **B** Volcano plot of DEGs in the old group; **C** Heatmap of DEGs in the young group; **D** Volcano plot of DEGs in the young group; **E–F** Venn diagram analysis of DEGs in different group (NC, normal control); **G** Histogram of DEGs in different group; **H**–**I** Heatmap of top 10 upregulated and downregulated DEGs in old and group; **J** Heatmap of regeneration-associated genes. (“Young vs NC”: young, 2-month-old mice, injury group vs the normal control group in GSE58982; “Old vs NC”:24-month-old mice, injury group vs the normal control group in GSE58982; “Injury vs NC”: injury group vs the normal control group in GSE96051)

To explore the differences in transcriptome changes after peripheral nerve injury in aged and young mice, we analyzed the dataset GSE58982 of the SNI model, which collected L4 ~ 5DRG 5 days after the operation. The boxplot and the expression density plots displayed that the distribution of values in the datasets is relatively consistent across all samples. The UMAP plots can reflect intra-group consistency and inter-group variability of the samples. Samples from different groups display circles of different colors. Samples within the group are clustered together, and samples from different groups are dispersed (Additional file 7: Figure S1). The datasets generated by GEO were normalized and met the quality requirements. Based on the comparative analysis between the surgery group and the normal control group of different age mice, we found that the number of DEGs of young mice (885: 475 upregulated and 410 downregulated) was relatively higher than that of old mice (693: 380 upregulated and 313 downregulated) (Additional file 1: Table S1). The distribution of two groups of DEGs is shown by the cluster heat map and volcano map (Fig. 2A–G).

As shown in Fig. 2H and Fig. 2I, the heat map shows the top 10 DEGs that are upregulated and downregulated. Among the 20 DEGs with the most significant changes in the two groups, 15 DEGs exist in the two groups, and the up-regulated DEGs are precisely the same. Venn diagram showed common or unique DEGs in old and young mice. There are 577 genes that appeared in the DEGs list in both old and young animals, and the expression trend of these DEGs is consistent in the two groups. None of the 577 shared genes that are upregulated in the old group are down-regulated in the young group, and vice versa, indicating that the higher intrinsic regeneration ability of young DRG neurons is mainly caused by their unique gene expression.

Gene transcription involved in peripheral axonal regeneration in response to injury has been extensively studied, from which a group of key genes associated with successful axon regeneration was known as “regeneration-associated genes” (RAGs) [34, 35]. The attenuated RAGs response induced by neuron-intrinsic injury is an essential reason for the failure of axon regeneration. Peripheral nerve injury involves the transcription of many RAGs, but the response between different ages has not yet been reported. Among the 25 common RAGs whose DRG expression levels changed after SNI [36, 37], 12 RAGs met our screening criteria. Interestingly, the vast majority of the 12 RAGs showed varying degrees of age dependence (Fig. 2J). For example, although Npy, Atf3, and Sprr1a are significantly upregulated after peripheral nerve injury in old and young mice, the up-regulated expression level in the young mice was higher than that in the old mice.

Taken together, there are both the same and differences in the transcriptional responses of the young and old DRGs induced by peripheral injury. Next, we will study the common axon regeneration program from 577 shared DEGs, and explore the age-dependent effects in their respective unique genes.

### Distinct functional enrichment in the old and young DRGs induced by SNI

**Fig. 3:**
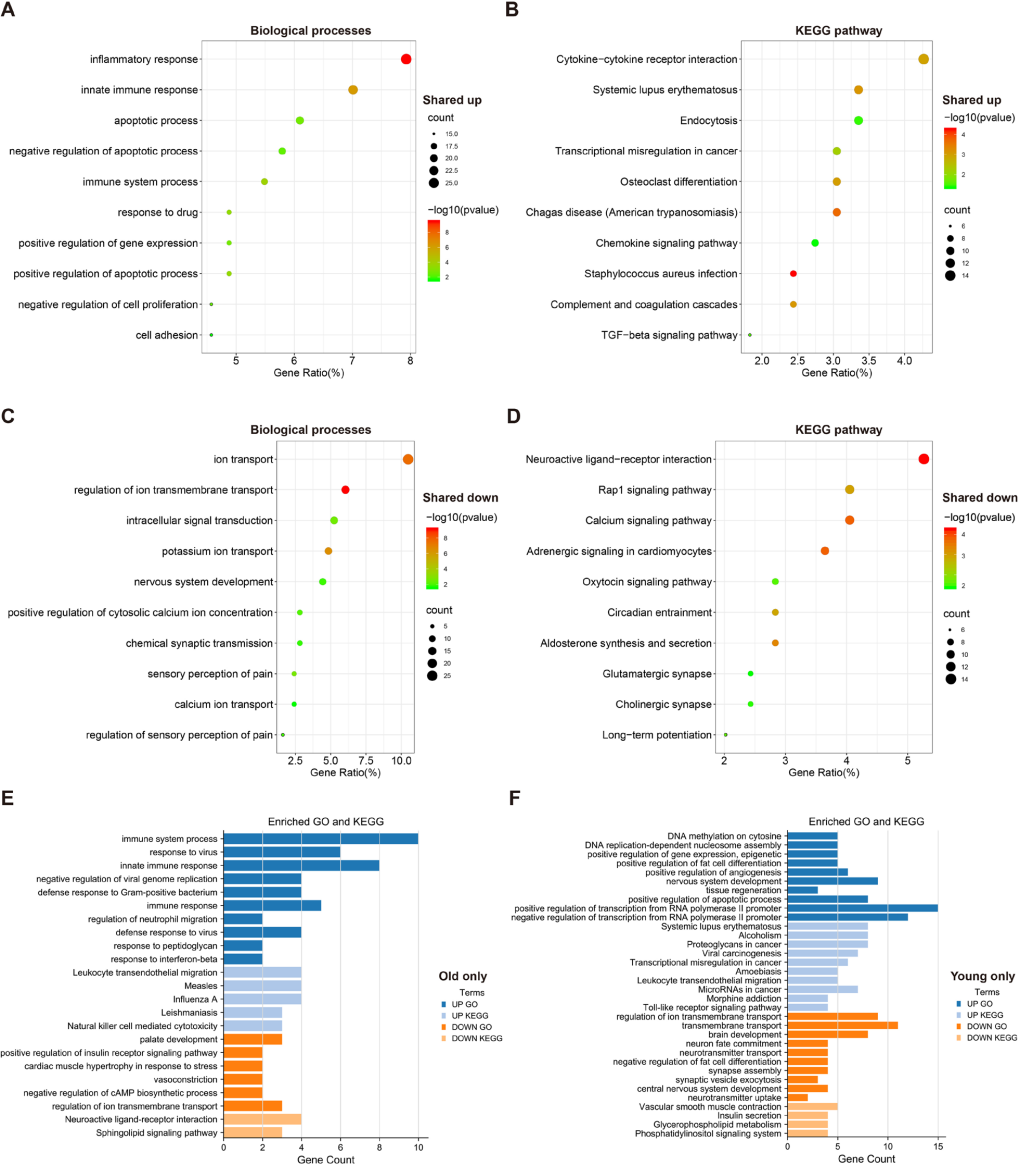
The GO analysis and KEGG pathway analysis of DEGs. **A** The GO analysis of shared upregulated DEGs; **B** The KEGG pathway analysis of shared upregulated DEGs; **C** The GO analysis of shared downregulated DEGs; **D** The KEGG pathway analysis of shared downregulated DEGs; **E** The GO analysis and KEGG pathway analysis of unique DEGs in the old group; **F** The GO analysis and KEGG pathway analysis of unique DEGs in the young group

Our transcriptome analysis revealed the two groups have a total of 577 common DEGs, with 116 unique DEGs in the old group and 308 unique DEGs in the young group. To assess the DEGs and their molecular function, we submitted the gene lists to the David database. We performed GO function enrichment analysis, providing strong inferences about its role in its biological processes (BP). Moreover, KEGG pathway analysis was also conducted, and the results are shown in Fig. 3. For shared up-regulated DEGs, many BP terms were closely related to the inflammatory response, immune system process, and apoptotic process (Fig. 3A). And the most enriched BP terms of shared down-regulated DEGs were associated with ion transport, chemical synaptic transmission, and sensory perception of pain (Fig. 3C, Additional file 2: Table S2); Furthermore, many BP terms were enriched in unique DEGs of the old mice: upregulated (immune system process, response to virus, and regulation of neutrophil migration) and downregulated (positive regulation of insulin receptor signaling pathway and negative regulation of cAMP biosynthetic process) (Fig. 3E, Additional file 4: Table S4). In unique DEGs of the young mice: upregulated (DNA methylation on cytosine, and positive regulation of gene expression, epigenetic) and downregulated (regulation of ion transmembrane transport, neurotransmitter transport, neurotransmitter uptake, and central nervous system development). Notably, both upregulated and downregulated DEGs are involved in regulating fat differentiation in young mice (Fig. 3F, Additional file 5: Table S5). According to the results of KEGG analysis, the most significantly involved pathways in shared up-regulated DEGs were Cytokine-cytokine receptor interaction, Chemokine signaling pathway, Complement and coagulation cascades, and TGF-beta signaling pathway (Fig. 3B). Consistent with the GO analysis results, the KEGG pathways in shared down-regulated DEGs were enriched, including Neuroactive ligand-receptor interaction, Calcium signaling pathway, Cholinergic synapse and Glutamatergic synapse (Fig. 3D, Additional file 3: Table S3). In unique DEGs of the old group, pathways showed enrichment in up-regulated (Leukocyte transendothelial migration and Natural killer cell mediated cytotoxicity) and down-regulated (Neuroactive ligand-receptor interaction and Sphingolipid signaling pathway) (Fig. 3E, Additional file 4: Table S4). However, in the young only group: up-regulated (Leukocyte transendothelial migration, Toll-like receptor signaling pathway) and down-regulated (Insulin secretion, Glycerophospholipid metabolism, and Phosphatidylinositol signaling system) (Fig. 3F, Additional file 5: Table S5).

### Establishment of the miRNA-TF-target gene network involved in conditioning lesion-induced transcriptional regulation

To investigate the potential roles of miRNAs, TF, and their regulated mRNA in the conditioning lesion model, we utilized the online TRRUST V.2 website to download the TF-gene regulatory relationships and downloaded the miRNA-mRNA interaction pairs from the ENCORI database. As shown in Additional file 8: Figure S2A, by intersecting the downloaded 827 TFs with 577 shared DEGs, we get 26 differentially expressed TFs. Using the GSE96051 dataset to verify the TF gene, 14 TFs marked with asterisks have statistical significance. Then, we predicted the filtered TFs. There were a total of 12 TF interacting with miRNAs, and 121 miRNA-mRNA interaction pairs were obtained by the ENCORI database. Moreover, we predicted 12 TF by TRRUST V.2 and obtained 214 pairs of TF-gene regulatory relationships. Next, integrating the miRNA-TF interaction pairs with TF-target gene pairs, a miRNA-TF-target gene interaction network was constructed (Additional file 8: Figure S2B, C).

The miRNA-TF-target gene network was analyzed and calculated using the "cytoHubba" plugin in Cytoscape. Those miRNAs with degree score ≥ 2 were regarded as hub miRNAs. The hub miRNA-TF relationship pairs are shown in Additional file 8: Figure S2D, from which it can be seen that miR-374b-5p impacts the 3 TF genes (Sox7, Nfkbiz, Nfil3), with the largest number of miRNAs interacting with Csf1.

### Construction of drug-hub gene network in condition-dependent regeneration

**Fig. 4:**
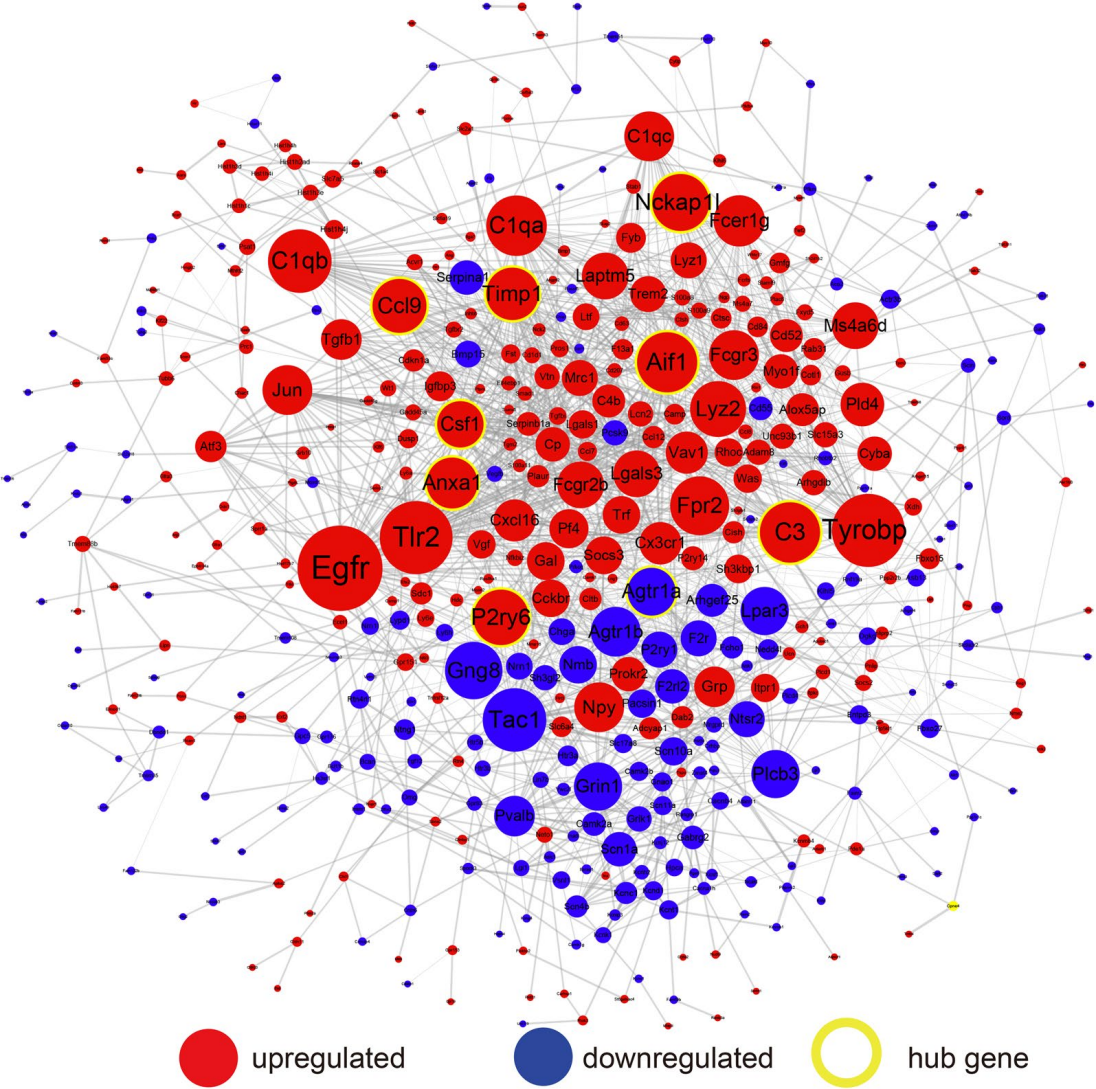
The protein–protein interaction (PPI) network. The red nodes mean upregulated shared DEGs, the blue nodes indicate downregulated shared DEGs and the yellow circles represent screened hub genes

**Fig. 5:**
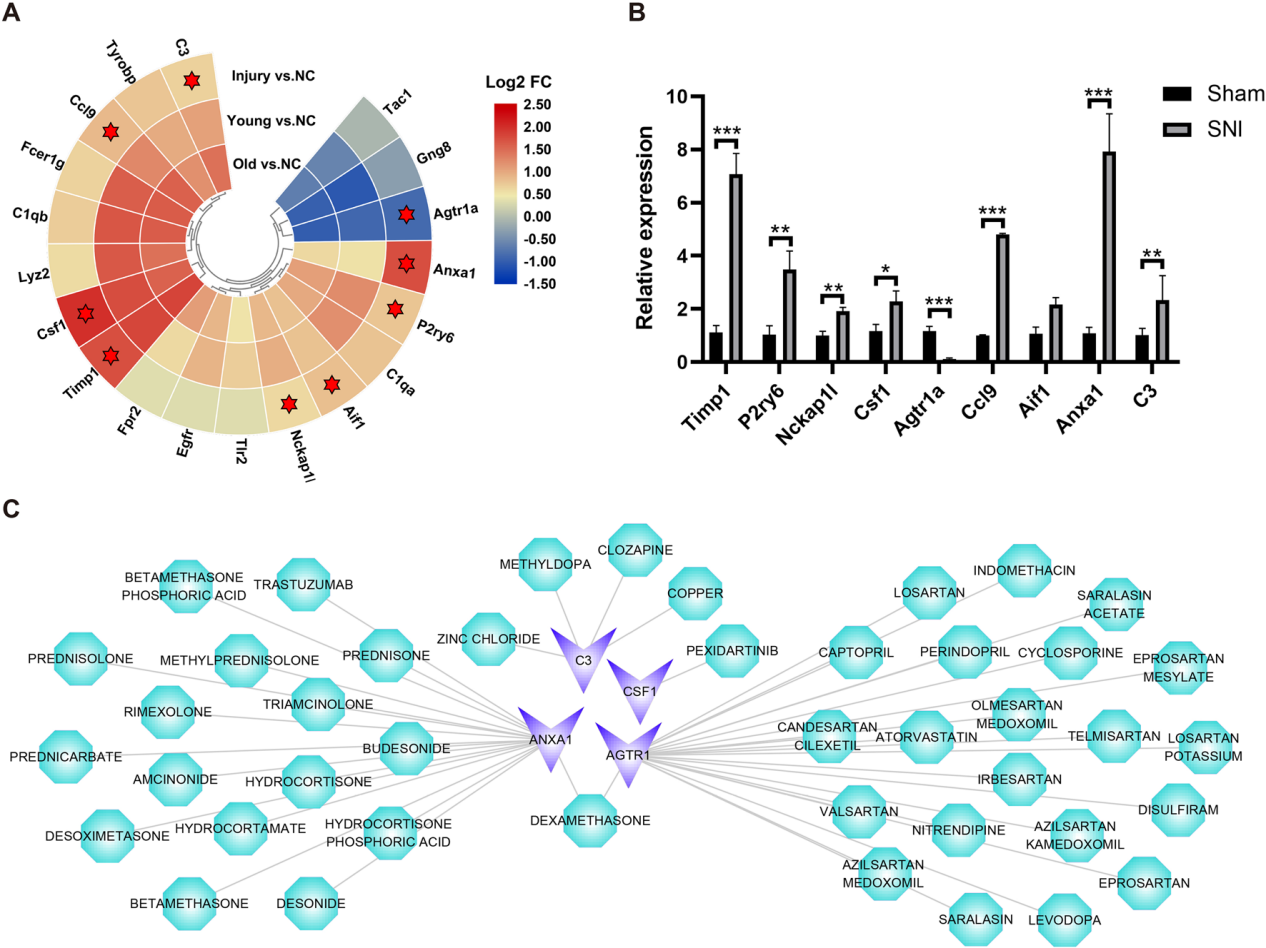
Drug prediction of hub genes. **A** Heatmap of hub genes with degree score ≥ 30, 9 hub genes marked with asterisks were identified by the GSE96051 dataset verified; **B** qRT-PCR showing the expression levels of hub genes (Sham and SNI groups, unpaired t-test, n = 6 mice/group). **C** In the drug-hub gene network, blue indicates hub genes and green indicates predicted drugs. (“Young vs NC”: young, 2-month-old mice, injury group vs the normal control group GSE58982; “Old vs NC”:24-month-old mice, injury group vs the normal control group in GSE58982; “Injury vs NC”: injury group vs the normal control group in GSE96051). *P < 0.05, **P < 0.01, ***P < 0.001

The PPI network constructed by shared DEG consists of 485 nodes and 1803 edges, among which 19 genes have degree values ≥ 30 (Fig. 4). After further verification in the GSE96051 dataset, 9 genes were selected as hub genes, including allograft inflammatory factor 1(Aif1), complement component 3(C3), NCK associated protein 1 like (Nckap1l), pyrimidinergic receptor P2Y, G-protein coupled, 6 (P2ry6), chemokine (C–C motif) ligand 9(Ccl9), tissue inhibitor of metalloproteinase 1(Timp1), annexin A1(Anxa1), angiotensin II receptor, type 1a(Agtr1a), colony stimulating factor 1 (macrophage) (Csf1) **(**Table 2**)**. Since sciatic nerve transection produces more thorough nerve damage than sciatic nerve crush, and both L4 and L5 DRGs are connected to the sciatic nerve, for subsequent experiments, we harvested L4 and L5 DRGs 5 days after sciatic nerve transection. The qRT-PCR results showed that Timp1, P2ry6, Nckap1l, Csf1, Ccl9, Anxa1, and C3 were upregulated in the SNI group while Agtr1a was downregulated in the SNI group compared to the normal control group, which are consistent with the expression trends of the GSE58982 and GSE96051 datasets, further verifying the quality of this dataset and the credibility of the analysis results **(**Fig. 5A, B**)**.

**Table 2:** Hub genes with higher scores

Gene	Gene symbol	Degree	Expression
Aif1	Allograft inflammatory factor 1	39	Up
C3	Complement component 3	39	Up
Nckap1l	NCK associated protein 1 like	37	Up
P2ry6	Pyrimidinergic receptor P2Y, G-protein coupled, 6	36	Up
Ccl9	Chemokine (C–C motif) ligand 9	35	Up
Timp1	Tissue inhibitor of metalloproteinase 1	34	Up
Anxa1	Annexin A1	33	Up
Agtr1a	Angiotensin II receptor, type 1a	31	Down
Csf1	Colony stimulating factor 1 (macrophage)	30	Up

Regarding 8 hub genes as potential therapeutic targets, 44 potential drugs that may affect the regeneration process were identified in the DGIdb database. Promising targets of these drugs included C3, ANXA1, AGTR1, and CSF1. As shown in Fig. 5C, 44 candidate drugs included C3 (zinc chloride, methyldopa, copper, and clozapine), ANXA1 (glucocorticoid drugs such as amcinonide, dexamethasone, hydrocortisone, methylprednisolone), AGTR1 (angiotensin II receptor antagonist such as irbesartan, eprosartan, azilsartan kamedoxomil, telmisartan) and CSF1 (pexidartinib). Among these drugs, dexamethasone simultaneously targeted ANXA1 and AGTR1. More experimental data are essential to confirm further the effect of these predicted drug candidates in promoting CNS repair after injury (Additional file 6: Table S6).

### Telmisartan enabled axon regeneration in SCI mice

**Fig. 6:**
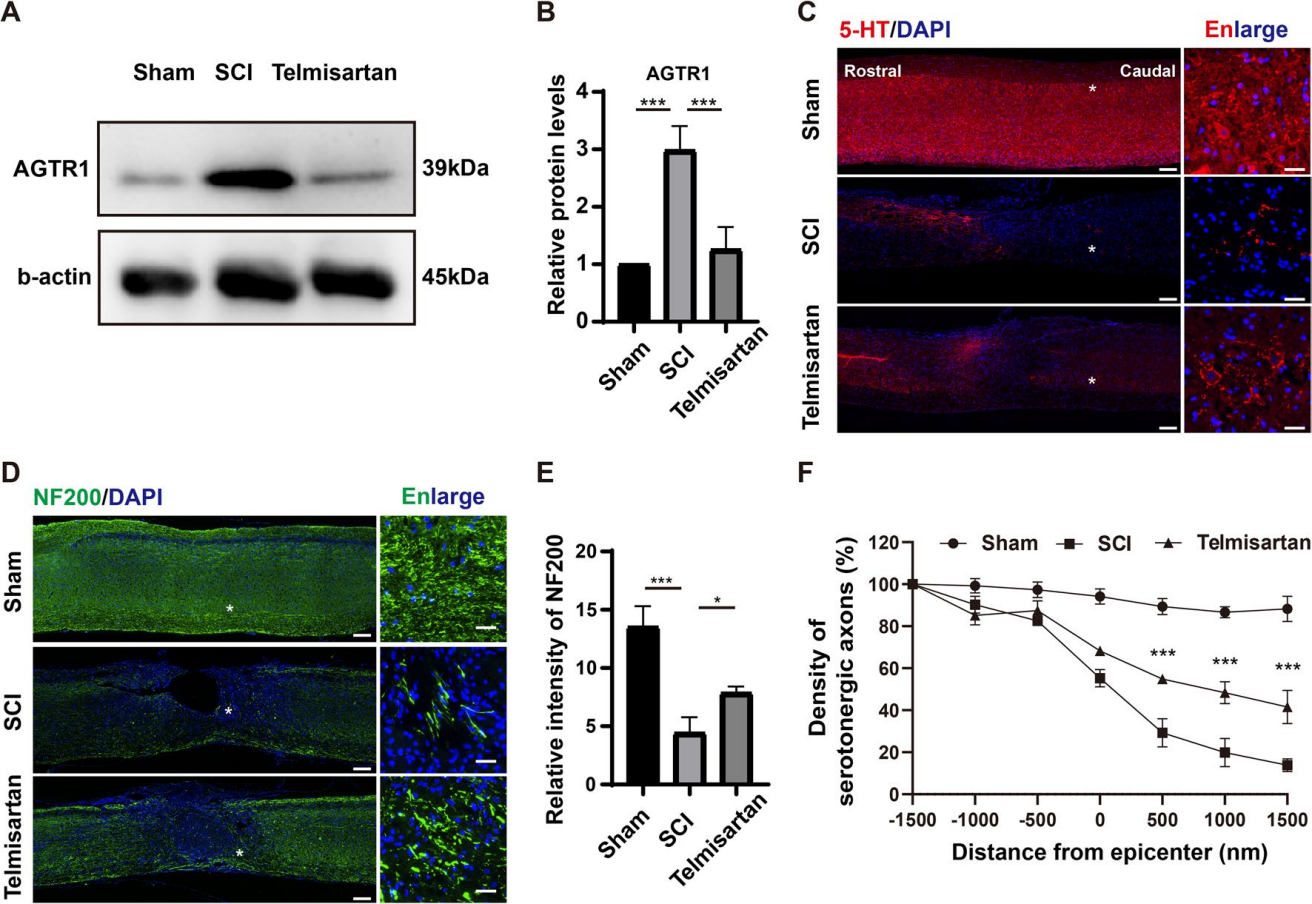
Telmisartan enabled axon regeneration after spinal cord contusion in mice. **A** Representative western blot image of the expressions of AGTR1 at 5 days after SCI. **B** Quantification of the expression of AGTR1 (normalized to levels in the Sham group) in spinal cord tissues in each group (n = 3, ***P < 0.001, one-way ANOVA followed by post hoc Tukey’s test). **C** Representative immunofluorescence images of serotonergic axons (5-HT, red) staining and DAPI (blue) in the sagittal view of the spinal cord. Bar = 200 µm and 25 µm (the enlarged images of the region of interest in the leftmost panorama). **D** Representative immunofluorescence images of NF200 (green) staining and DAPI (blue) in the sagittal view of the spinal cord. Bar = 200 µm and 25 µm (the enlarged images of the region of interest in the leftmost panorama). **E** Quantification of the NF200-positive intensity in the injured center (n = 3, *P < 0.05, ***P < 0.001, one-way ANOVA followed by post hoc Tukey’s test). **F** Quantification of serotonergic axons at an indicated distance beyond the lesion (n = 3, ***P < 0.001, two-way ANOVA followed by post hoc Tukey’s test, * denominated as the comparison between the SCI group and the telmisartan group)

As the only downregulated gene in the hub genes after conditioning lesion, AGTR1 and its target drug telmisartan greatly attracted our attention. Previous studies have shown that telmisartan is strongly neuroprotective and neurorestorative by blocking the angiotensin II type 1 receptor (AGTR1) after traumatic brain injury [38]. We next examined the effect of telmisartan on key proteins AGTR1 at 5 days after SCI (Fig. 6A). At 5 days post-injury, western blot results showed that AGTR1 expression was increased in the SCI group compared to the sham group, whereas the telmisartan group showed a significantly downregulated AGTR1 level compared with the SCI group (Fig. 6B). We further examined the effect of telmisartan on axon regeneration by immunofluorescence staining at 6 weeks after SCI (Fig. 6C, D). Our results showed that telmisartan intervention effectively increased NF200-positive neurons around the injury area (Fig. 6E). Moreover, less than 15% of 5-HT positive axons extended to 1500 nm in the SCI group, notably, the telmisartan-treated group showed longer-distance elongation of 5-HT positive axons with 41% of axons extending to 1500 nm (Fig. 6F). Taken together, telmisartan enables axonal regeneration by inhibiting the expression of AGTR1 after SCI.

### Telmisartan improved the sensory and motor function recovery of mice after SCI

**Fig. 7:**
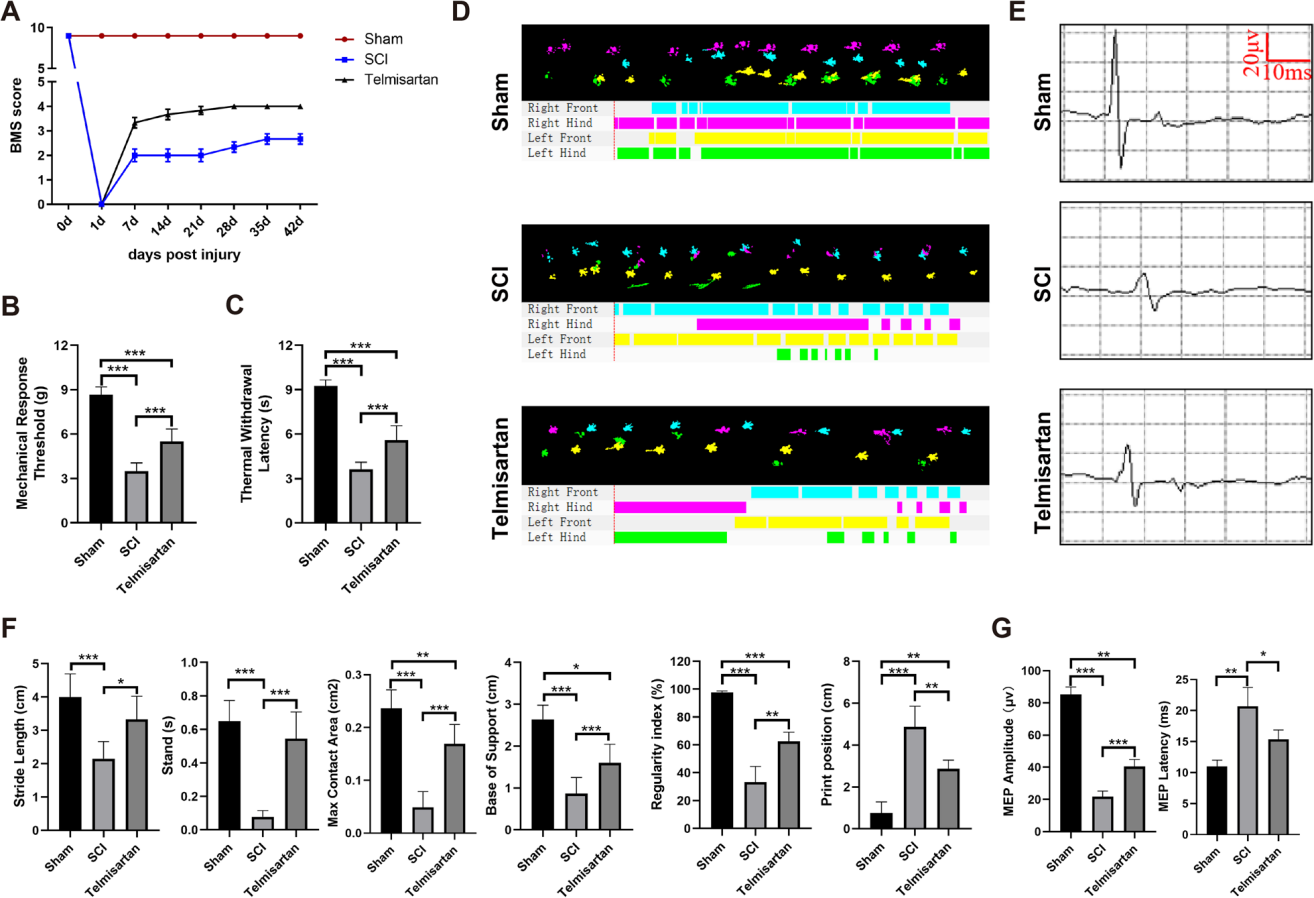
Therapeutic effects of telmisartan on behavioral function after mice SCI. **A** Basso Mouse Scale (BMS) scoring (two-way analysis of variance with Tukey’s post hoc test, n = 6 mice/group); **B**, **C** The sensitivities measure to mechanical and thermal stimulation (one-way analysis of variance with Tukey’s post hoc test, n = 6 mice/group); **D** Catwalk automated quantitative gait analysis show print view and timing view of sham group, SCI group, and telmisartan group (n = 6 mice/group); **E** Electrophysiology show motor evoked potential (MEP) (n = 6 mice/group); **F** stride length (cm), stand (s), base of support (cm), max contact area (cm^2^), regularity index (%), and print position (cm) of catwalk parameters analysis (one-way analysis of variance with Tukey’s post hoc test, n = 6 mice/group); **G** quantification of amplitude (uV) and latency (ms) of MEP (one-way analysis of variance with Tukey’s post hoc test, n = 6 mice/group). *P < 0.05; **P < 0.01; ***P < 0.001

To assess the effectiveness of telmisartan on SCI, telmisartan was administered by oral gavage for 2 consecutive weeks after the mice injury. BMS scoring showed that the telmisartan group significantly improved scores compared with the SCI group, revealing a restorative effect of telmisartan on decreased motor function (Fig. 7A). In addition, the mechanical response threshold and thermal withdrawal latency were evaluated to test neuropathic pain sensitivity in mice exposed to SCI. As shown in Fig. 7B and Fig. 7C, SCI caused a decrease in withdrawal latency of mechanical and thermal sensitivity compared with sham values, traditionally interpreted as hyperalgesia. However, telmisartan could reduce hyperalgesia, which means that telmisartan could ameliorate the sensory function recovery of mice after spinal cord injury. Furthermore, catwalk automated quantitative gait analysis and electrophysiological analysis were carried out to further detect the improvement of sensory and motor functions. Catwalk automated quantitative gait analysis has a similar behavioral recovery situation with previous tests (Fig. 7D). Our data showed that the telmisartan group significantly improved the stand, max contact area, stride length, base of support, and regularity index compared to the SCI group. In addition, compared with the sham group, the printing position in the SCI group increased significantly, while that in the telmisartan group decreased. Notably, there was no significant difference in the stride length and standing time between the telmisartan group and the sham group (Fig. 7F). To evaluate the electrophysiological outcomes of telmisartan treatment, MEPs were recorded at 6 weeks after SCI (Fig. 7E). Relative to the results of the SCI group, the latency of the MEP was shortened, and the amplitude was improved in the telmisartan group (Fig. 7G). All above confirms that telmisartan significantly promoted the recovery of sensory and motor function after SCI in mice.

## Discussion

Conditioning lesion triggers a cascade of events in DRG that result in the transformation of DRG neurons into active growing cells after axotomy, which promotes their central axon branches regeneration after SCI [39]. Numerous studies have identified targets for promoting axonal regeneration in the spinal cord through conditioned injury, such as the elevation of cyclic AMP and activation of STAT3 [3, 9, 40, 41]. However, the molecular mechanism of DRG neuron axon regeneration after peripheral nerve injury remains largely unknown. In the current study, we bioinformatically identified transcriptome change in DRG neurons in old and young injured DRG neurons during the early stages of conditioning lesion. We firstly constructed the miRNA-TF-target gene network to reveal the regulatory effect of miRNA on TFs in the regenerative transcription response after conditional injury. Then, we identified hub genes, including C3, ANXA1, AGTR1, CSF1, and their predicted drug to construct a drug-hub gene network. Telmisartan, an AGTR1 antagonist selected from the drug-hub gene network in condition-dependent regeneration, validated the effectiveness in the mice SCI model. Unlike classical study paradigms, we discovered an improved platform for repairing SCI from the mechanism of conditioning lesion.

Inflammatory and immune responses following CNS injury are the main factors leading to secondary tissue damage. The pathological aggregation and proliferation of various cells make the microenvironment full of various mediators, including pro-inflammatory chemokines/cytokines and reactive oxygen species [42, 43]. Damage to the peripheral nervous system involves multiple immune cells, including macrophages, dendritic cells, T cells, and B cells. The persistent high inflammatory response is a culprit in age-related regeneration disorders of the peripheral nerve [44, 45]. Based on these findings, we investigated whether the dysfunctional genes of the DRGs produced similar functional changes after SNI. In this study, we identified 327 upregulated genes and 250 downregulated genes, which maintained the same trend in both the old group and the young group. Subsequently, we carried out functional enrichment analysis on the upregulated and downregulated DEGs, respectively. Consistent with previous studies, the transcriptional analysis results showed that the differentially upregulated genes included those related to inflammation and immune response. In contrast, the downregulated DEGs included those encoding ion transport and synaptic transmission-related genes.

It is worth noting that from the results of functional enrichment analysis, some C–C motif chemokine ligand genes (CCL12, CCL9, CCL8, CCL7) are significantly up-regulated in the inflammatory response, and some complement component genes (C1QB, C1QA, C3, C4B, C1QC) are significantly up-regulated in the immune response. In addition, these C–C motif chemokine ligand genes participate in cytokine-cytokine receptor interaction and chemokine signaling pathway, and complement component genes participate in complement and coagulation cascades. Moreover, CSF1, HMGB2, C3, ANXA1, S100A9, S100A8, and TLR2 are all involved in regulating both inflammation and immune response, of which C3, ANXA1, and CSF1 are the hub genes we selected. As the convergence point of immune activation of the classical complement pathway, alternative complement pathway, and lectin pathway, complement C3 plays an indispensable part in the recruitment and activation of inflammatory cells, the opsonization of pathogens, and the phagocytosis of cell debris [46, 47]. Anderson et al. revealed that complement proteins such as C3 are upregulated after SCI, and complement protein C3 has a negative regulatory effect on axonal growth and neuronal survival. However, compared with WT mice, C3 deficient mice promoted the regeneration of sensory fibers in the conditioned injury model to regenerate the SCI lesion area [48].

The genes encoding downregulation of ion transport are mainly related to voltage-gated channels (KCNG4, KCNC1, KCNMB1, KCNH2, KCNF1, KCND1, KCNJ12, CACNA1H, CACNB4). In the vertebrate nervous system, voltage-gated potassium channels regulate neurotransmitter transport, affecting channel activity and neuronal excitability. KCNQ2/3 channels can control the release of glutamate and γ-aminobutyric acid (GABA) to inhibit neuronal excitability. Abnormal GABAergic synaptic transmission was found in KCNQ2 gene-deficient mice [49]. After an axonal injury, the formation of new growth cones relies heavily on calcium influx. Axons will fail to regenerate fresh growth cones, if under the environment of calcium-free [50]. A short burst of action potential occurs when the peripheral axons of DRG neurons are severed, but due to the decrease of sensory input, their subsequent electrical activity will decrease. This reduction in electrical activity seems to increase the long-term regeneration of DRG axons [51]. The voltage-gated calcium channel subunit Alpha2delta2, by the inflow of calcium ions through the Cav2 channel, controls the growth of axons during the development and the regeneration of axons in the after CNS injury [2]. The increased expression of a2d2 in corticospinal neurons leads to the loss of axon regeneration ability during development stage and after SCI. By administrating a2d2 gabapentin pharmacological blocker (gabapentin) can promote the plasticity and regeneration of the corticospinal axon structure [52, 53].

According to the results of functional enrichment analysis of unique DEGs in the old group and the young group, the unique DEGs in the old group are mainly enriched in the immune response (SLPI, OAS2, MYD88), and participate in the regulation of immune-related pathways (RAC2, PIK3CG) such as leukocyte transendothelial migration, natural killer cell mediated cytotoxicity. However, these unique DEGs in the young group were mainly enriched in ion transmembrane transport (KCNV1, KCNB1, KCNA2, CACNA2D), neurotransmitter transport and uptake (SV2B, SV2A), and involved in the regulation of lipid metabolism pathways (DGKZ, DGKH, CDS2) such as glycerophospholipid metabolism, phosphatidylinositol signaling system. Interestingly, among the unique DEGs in the young group, some clustered histone genes (HIST2H3B, HIST1H4K, HIST1H4N, HIST2H3C1, HIST1H4F) are up-regulated, mainly enriched in DNA methylation on cytosine, DNA replication-dependent nucleosome assembly and positively regulate gene expression from epigenetics. Several studies have reported either global DNA methylation status, or demethylation is required for the pre-conditioned injury model. However, how these clustered histone genes regulate methylation of the cytosine base at CpG sites in DRGs still needs further study [54]. Previous studies have shown that histone acetylation mediated by Creb-binding protein (Cbp) can promote the regeneration and sprouting of mouse proprioceptive DRG neurons [55]. Moreover, after PNS injury, administration of HDAC1/2 inhibitor can delay histone deacetylase to promote the transformation of Schwann cells into a repair state and accelerate peripheral nerve regeneration [56]. Functional enrichment in both young and old mice indicated that the potentional mechanism of aging-dependent regenerative decline. Compared with the young mice, the old mice produced a more robust immune response in the DRG after SNI. In contrast, regulation of ion transport, neurotransmitter transport and uptake, and epigenetic regulation of gene expression, such as DNA methylation, was identified in the young mice.

In view of the fact that less than 2% of the transcripts encode proteins, MicroRNA, as a critical epigenetic regulator, plays a vital role in regulating biological processes such as neuronal development, neuronal plasticity, and axon regeneration by inhibiting translational progress or inducing mRNA degradation [57]. Transcription factors play a pivotal role in the gene regulatory network, and they inhibit or enhance target gene expression by regulating the transcriptional process of target genes. And many TFs are involved in the RAGs activated after conditional injury [34]. However, how the miRNAs regulate TFs to affect the expression of target genes remains to be explored. To this end, we built the miRNA-TF-target network and screened out the hub miRNA-TF relationship pairs. Previous researches have reported that suppression of miR-17-5p promotes axonal regeneration of cortical neurons and neurite outgrowth of DRG sensory neurons through miR-17-5p/STAT3/GAP-43 pathway [58, 59]. MiR-22-3p has also been demonstrated to upregulate and regulate the MAPK pathway after spinal cord ischemia–reperfusion [60]. Circular RNA circ-Ankib1 was downregulated after SNI, which induced Schwann cells proliferation and axon regeneration by sponging miR-423-5p [61]. In general, these screened miRNAs have been confirmed to affect the process of axonal regeneration, but their effect on TF still needs to be verified.

The DGIdb database makes it possible to find therapeutic agents for axon regeneration through drug-gene relationships. ANXA1, as a glucocorticoid medium, has been shown to reduce inflammatory responses and produce neuroprotective effects by inhibiting phospholipase A2 activation in the rat SCI model. C3, ANXA1, and CSF1 are involved in regulating inflammation response [62]. Zinc chloride was presented as a reactant, and has a concentration-dependent inhibition and enhancement effect on guinea pig complement component activity in vitro [63]. Pexidartinib could regulate the myelin environment by inducing microglial ablation, thereby preserving myelination in MS models [64, 65]. Although zinc chloride and pexidartinib have not been reported to promote axon regeneration, based on the interaction with C3 and CSF1, we speculated that the two drugs could reduce inflammation, thus promoting axon regeneration and protecting neurons. AGTR1 encodes angiotensin II type 1 receptor (AT1R) protein, which is an indispensable part of the renin-angiotensin system [66]. Immune profiling revealed that AGTR1 is a hallmark gene in Parkinson’s disease (PD) and that its antagonists have been shown to reduce AT1R upregulation, inducing dopaminergic (DA) neuronal cell death and nigrostriatal dysfunction [67]. However, compared with the matched controls, AT1R expression in substantia nigra DA neurons is significantly down-regulated during PD progression. The contradictory results between research and clinical phenomena indicate the complexity of AGTR1 in regulating the pathological process of PD, which further attracts us to explore whether AGTR1 downregulation has neuroprotective effects in the CNS. According to previous studies, AT1R antagonists, angiotensin II receptor blockers (ARBs) or sartans have neuroprotective effects after stroke, inflammatory brain, neurodegenerative diseases, and brain injury [38, 68–70]. These two angiotensin II receptor antagonists adjust two signal pathways, blockade of AGTR1 and activation of the peroxisome proliferator-activated receptor gamma (PPARγ, encoded by PPARG), thereby protecting cerebral blood flow and reducing inflammation [38]. Mounting evidence has shown that AT1R antagonists have neuroprotective effects in animal models of neurodegeneration or traumatic brain diseases, but their application in spinal cord injury still needs further exploration [71]. Therefore, we selected telmisartan, a clinically common AT1R antagonist, for treatment in a mouse spinal cord contusion model. Our results showed that telmisartan promoted axonal regeneration by inhibiting the expression of AGTR1 after SCI. Previous studies have shown that telmisartan inhibits AGTR1 gene expression through PPARγ activation and the AGTR1 downregulation is PPARγ dependent. The AT1R binding effect was observed at lower concentrations of telmisartan, whereas at higher concentrations a dual effect of AT1R binding and AT1R downregulation occurred [72]. Telmisartan may inhibit AGTR1 gene expression in SCI through PPARγ activation, but the exact mechanism needs further experimental verification. Extensive preclinical studies are still necessary to determine the specific molecular mechanism, dose–response, and therapeutic window of telmisartan for SCI. The recovery of sensory and motor function after mice SCI fully proved the credibility of our discovery of therapeutic targets for SCI based on molecular mechanisms of axon regeneration after conditioning lesion.

In summary, we provided a transcriptome profiling study for revealing the potential molecular mechanisms of young and old mice DRGs following conditional injury. Our findings suggested the identified hub genes, including C3, ANXA1, AGTR1, CSF1, and their related drug, may affect the axonal regeneration program. In addition, the results of this study showed that telmisartan promoted axonal regeneration by inhibiting the expression of AGTR1 after SCI. In this way, we provided a successful paradigm for identifying therapeutic targets for CNS based on molecular mechanisms of DRG axon regeneration after conditioning lesion.

## Supplementary Information

**Additional file 1: Table S1.** The list of DEGs.

**Additional file 2: Table S2.** GO analysis of shared DEGs.

**Additional file 3: Table S3.** KEGG analysis of shared DEGs.

**Additional file 4: Table S4.** GO and KEGG analysis of DEGs in the old only group.

**Additional file 5: Table S5.** GO and KEGG analysis of DEGs in the young only group.

**Additional file 6: Table S6.** Predicted drugs of hub genes by DrugBank.

**Additional file 7: Figure S1.** The gene expression data quality of GSE58982 and GSE96051 datasets. **A** The boxplots of the GSE58982 dataset. The abscissa represents the names of samples, and the ordinate represents the normalized expression levels. **B** The UMAP plot of the GSE58982 dataset shows the distribution relationships between the samples. **C** The density map of the GSE58982 dataset shows the expression of each sample. **D** The boxplots of the GSE96051 dataset. (E) The UMAP plot of the GSE96051 dataset. **F** The density map of the GSE96051 dataset.

**Additional file 8: Figure S2.** Construction of the miRNA-TF-target gene Network. **A** Venn diagram to obtain shared differentially expressed TFs (DEG, differentially expressed gene); **B** Heatmap of shared differentially expressed TFs; the differentially expressed TFs marked with asterisks were identified by the GSE96051 dataset verified, and the TFs marked with a white asterisk does not predict the targeted miRNA. **C** The miRNA-TF-target gene Network, blue indicates miRNAs, green indicates TFs, pink indicates mRNAs; **D** The hub miRNA-TF relationship pairs. (“Young vs NC”: young, 2-month-old mice, injury group vs the normal control group GSE58982; “Old vs NC”:24-month-old mice, injury group vs the normal control group in GSE58982; “Injury vs NC”: injury group vs the normal control group in GSE96051).

## Data Availability

The raw datasets generated for this study can be found in the Gene Expression Omnibus (GEO) (http://www.ncbi.nlm.nih.gov/geo/), and the GEO accession numbers are GSE58982 and GSE96051.

## Abbreviations

DRG: Dorsal root ganglia
CNS: Central nervous system
SCI: Spinal cord injury
SNI: Sciatic nerve injury
DEGs: Differentially expressed genes
GO: Gene ontology
KEGG: Kyoto Encyclopedia of Genes and Genomes
PPI: Protein–protein interaction
