# Supplementary material for: Discovery of therapeutic targets for spinal cord injury based on molecular mechanisms of axon regeneration after conditioning lesion
Source: J Transl Med. 2023 Jul 28;21:511. doi: 10.1186/s12967-023-04375-1 (PMC10385911; doi:10.1186/s12967-023-04375-1)
Supplement: Supplementary file 2 — Additional file 2: Table S2. GO analysis of shared DEGs. [file 12967_2023_4375_MOESM2_ESM.docx]

**Table S2. GO analysis of shared DEGs**

| **Term** | **Count** | **P-Value** | **Genes** |
| --- | --- | --- | --- |
| **Upregulated** |  |  |  |
| inflammatory response | 26 | 3.40E-10 | UCN, CCL12, CSF1, HMGB2, FPR2, AIF1, C3, C4B, NDST1, CCL9, CCL8, CCL7, NFKBIZ, STAB1, SMAD1, TGFB1, ANXA1, CYBA, GAL, SDC1, ADAM8, S100A9, CHST2, S100A8, TLR2, PF4 |
| innate immune response | 23 | 5.54E-07 | C1QB, C1QA, CD84, FCER1G, ANXA1, CSF1, UNC93B1, HMGB2, CYBA, TREM2, C3, C4B, LGALS3, CD1D1, TYROBP, LCN2, ANG, SH2D1B1, S100A9, S100A8, CAMP, C1QC, TLR2 |
| apoptotic process | 20 | 2.18E-03 | SH3GLB1, CSRNP1, SEMA6A, TNFRSF12A, SH3KBP1, ITPR1, GADD45G, TGFBR2, DAB2, LGALS1, CCKBR, PPP2R2B, CASP6, WT1, LCN2, PDCD2, CHAC1, PHLDA1, S100A9, S100A8 |
| negative regulation of apoptotic process | 19 | 4.69E-03 | UCN, JUN, CDKN1A, DUSP1, PLAUR, NME2, AIF1, EGFR, FAM129B, PLAC8, LGALS3, SOCS3, DAB2, WT1, RPS6KA1, CTSH, NCKAP1L, TIMP1, LTF |
| immune system process | 18 | 1.60E-04 | C1QB, C1QA, SEMA4A, CD84, ANXA1, CSF1, UNC93B1, HMGB2, C3, LGALS3, CD1D1, LCN2, SH2D1B1, S100A9, S100A8, C1QC, LTF, TLR2 |
| positive regulation of apoptotic process | 16 | 3.54E-04 | JUN, TGFB1, ANXA1, TNFRSF12A, GADD45A, DUSP1, IGFBP3, GADD45G, GAL, CASP6, WT1, TSPO, PDCD2, NUPR1, PHLDA1, TGM2 |
| response to drug | 16 | 4.00E-04 | APOBEC1, SMAD1, JUN, CDKN1A, TGFB1, ANXA1, HMGB2, CYBA, SLC6A4, TGFBR2, GAL, LGALS1, LCN2, TSPO, PLIN2, NCKAP1L |
| positive regulation of gene expression | 16 | 2.06E-03 | SMAD1, UCN, TGFB1, CSF1, DTNBP1, PLAUR, HMGB2, SLC6A4, ADCYAP1, NFIL3, LCN2, CTSH, REG1, ATF3, PF4, TLR2 |
| negative regulation of cell proliferation | 15 | 3.77E-03 | SMAD1, JUN, CDKN1A, BTG1, TGFB1, IGFBP3, ADARB1, TGFBR2, GAL, WT1, HIST1H2AD, REG1, SOX7, SKAP2, TLR2 |
| cell adhesion | 15 | 2.50E-02 | CX3CR1, CD63, CD84, LAMB3, TNFRSF12A, GP1BB, WISP1, CLDN11, VTN, SDK1, FLRT3, CHL1, STAB1, ITGA7, TGFBI |
| **Downregulated** |  |  |  |
| ion transport | 26 | 2.66E-08 | KCNG4, KCNC1, GRIK1, CACNA1H, KCNT1, SCN11A, KCNMB1, SLC22A18, SLC17A8, ASIC1, SCN1A, KCNH2, KCNF1, KCND1, KCNJ12, KCNIP3, HTR3A, HTR3B, GABRG2, GRIN1, SCN10A, CACNB4, KCNS1, FXYD2, KCNK1, SCN4B |
| regulation of ion transmembrane transport | 15 | 7.02E-10 | KCNH2, KCNF1, KCNG4, KCND1, KCNC1, KCNJ12, KCNIP3, NEDD4L, CACNA1H, SCN10A, CACNB4, KCNS1, SCN11A, SCN4B, SCN1A |
| intracellular signal transduction | 13 | 2.85E-03 | DGKG, CCDC68, DAPK2, ASB13, RASGRP1, PLCB3, RIT2, PPP1R1A, PPP1R1C, RASSF5, ARHGEF4, PRKCQ, PLCD4 |
| potassium ion transport | 12 | 3.15E-07 | KCNH2, KCNF1, KCNT1, KCND1, KCNG4, KCNC1, KCNS1, KCNJ12, FXYD2, KCNMB1, KCNIP3, KCNK1 |
| nervous system development | 11 | 1.42E-02 | NTNG1, CAMK2B, NRN1, AMIGO3, NRN1L, CNTN6, GNG8, CHD5, GRIK1, INSM1, NEUROG3 |
| positive regulation of cytosolic calcium ion concentration | 7 | 8.23E-03 | NMB, GPR35, P2RY1, F2R, TAC1, AGTR1A, AGTR1B |
| chemical synaptic transmission | 7 | 1.63E-02 | PLCB3, KCNMB1, TAC1, FGF12, HTR5B, GABRG2, GRIN1 |
| sensory perception of pain | 6 | 2.15E-03 | SCN10A, KCNIP3, P2RY1, TAC1, CACNA1H, GRIN1 |
| calcium ion transport | 6 | 2.58E-02 | CAMK2B, CACNB4, CAMK2A, CACNA1H, GRIN1, ASIC1 |
| regulation of sensory perception of pain | 4 | 1.24E-02 | TMEM100, SCN11A, F2R, ACPP |
