# Supplementary material for: Discovery of therapeutic targets for spinal cord injury based on molecular mechanisms of axon regeneration after conditioning lesion
Source: J Transl Med. 2023 Jul 28;21:511. doi: 10.1186/s12967-023-04375-1 (PMC10385911; doi:10.1186/s12967-023-04375-1)
Supplement: Supplementary file 3 — Additional file 3: Table S3. KEGG analysis of shared DEGs. [file 12967_2023_4375_MOESM3_ESM.docx]

**Table S3. KEGG analysis of shared DEGs**

| **Term** | **Count** | **P-Value** | **Genes** |
| --- | --- | --- | --- |
| **Upregulated** |  |  |  |
| Cytokine-cytokine receptor interaction | 14 | 2.66E-08 | ACVR1, CX3CR1, CCL12, TGFB1, TNFRSF12A, CSF1, INHBB, TGFBR2, CXCL16, CCL9, CCL8, CCL7, IL13RA1, PF4 |
| Systemic lupus erythematosus | 11 | 7.02E-10 | C1QB, C3, C4B, C1QA, HIST1H2AD, HIST1H4H, HIST1H4I, HIST1H4J, HIST1H3D, C1QC, HIST1H3E |
| Endocytosis | 11 | 2.85E-03 | SH3GLB1, DAB2, RAB31, SH3KBP1, PIP5KL1, CHMP1B, CLTB, WAS, EGFR, TGFBR2, RAB11FIP5 |
| Chagas disease | 10 | 3.15E-07 | C1QB, C3, C1QA, CCL12, JUN, TGFB1, PPP2R2B, TGFBR2, C1QC, TLR2 |
| Osteoclast differentiation | 10 | 1.42E-02 | SOCS3, FCGR3, JUN, TYROBP, TGFB1, CSF1, CYBA, TREM2, FCGR2B, TGFBR2 |
| Transcriptional misregulation in cancer | 10 | 8.23E-03 | SMAD1, CDKN1A, WT1, IGFBP3, NFKBIZ, ASPSCR1, NUPR1, HIST1H3D, TGFBR2, HIST1H3E |
| Chemokine signaling pathway | 9 | 1.63E-02 | CX3CR1, CCL9, CCL12, CCL8, CCL7, WAS, VAV1, CXCL16, PF4 |
| Staphylococcus aureus infection | 8 | 2.15E-03 | C1QB, C3, C4B, C1QA, FCGR3, FPR2, FCGR2B, C1QC |
| Complement and coagulation cascades | 8 | 2.58E-02 | C1QB, C3, C4B, C1QA, PROS1, PLAUR, F13A1, C1QC |
| TGF-beta signaling pathway | 6 | 1.24E-02 | ACVR1, SMAD1, TGFB1, FST, INHBB, TGFBR2 |
| **Downregulated** |  |  |  |
| Neuroactive ligand receptor interaction | 13 | 1.22E-04 | GPR35, F2R, GPR83, GRIK1, LPAR3, HTR5B, GABRG2, AGTR1A, AGTR1B, GRIN1, P2RY1, F2RL2, NTSR2 |
| Calcium signaling pathway | 10 | 2.59E-04 | CAMK2B, PLCB3, CAMK2A, F2R, PLCD4, HTR5B, CACNA1H, AGTR1A, AGTR1B, GRIN1 |
| Adrenergic signaling in cardiomyocytes | 9 | 2.59E-04 | CAMK2B, PLCB3, CACNB4, PPP1R1A, FXYD2, CAMK2A, SCN4B, AGTR1A, AGTR1B |
| Aldosterone synthesis and secretion | 7 | 5.11E-04 | CAMK2B, PLCB3, CAMK2A, CACNA1H, CAMK1G, AGTR1A, AGTR1B |
| Rap1 signaling pathway | 10 | 9.16E-04 | GNAO1, PLCB3, RASSF5, MAGI3, P2RY1, F2R, VEGFB, LPAR3, FGF12, GRIN1 |
| Circadian entrainment | 7 | 1.02E-03 | CAMK2B, GNAO1, PLCB3, CAMK2A, GNG8, CACNA1H, GRIN1 |
| Long-term potentiation | 5 | 7.56E-03 | CAMK2B, PLCB3, PPP1R1A, CAMK2A, GRIN1 |
| Oxytocin signaling pathway | 7 | 8.52E-03 | CAMK2B, GNAO1, PLCB3, CACNB4, KCNJ12, CAMK2A, CAMK1G |
| Cholinergic synapse | 6 | 1.07E-02 | CAMK2B, GNAO1, PLCB3, KCNJ12, CAMK2A, GNG8 |
| Glutamatergic synapse | 6 | 1.15E-02 | GNAO1, PLCB3, GNG8, GRIK1, SLC17A8, GRIN1 |
