# Supplementary material for: Discovery of therapeutic targets for spinal cord injury based on molecular mechanisms of axon regeneration after conditioning lesion
Source: J Transl Med. 2023 Jul 28;21:511. doi: 10.1186/s12967-023-04375-1 (PMC10385911; doi:10.1186/s12967-023-04375-1)
Supplement: Supplementary file 4 — Additional file 4: Table S4. GO and KEGG analysis of DEGs in the old only group. [file 12967_2023_4375_MOESM4_ESM.docx]

**Table S4. GO and KEGG analysis of DEGs in the old only group**

| **Term** | **Count** | **P-Value** | **Genes** |
| --- | --- | --- | --- |
| **Upregulated GO** |  |  |  |
| immune system process | 10 | 2.88E-07 | IFITM3, IFITM2, SLPI, OAS2, CLEC4N, MX2, PRG2, PGLYRP1, MYD88, PIK3CG |
| response to vrus | 6 | 1.81E-06 | IFITM3, IFITM2, OAS2, MX2, IRAK3, MYD88 |
| innate immune response | 8 | 4.65E-05 | IFITM3, IFITM2, SLPI, OAS2, CLEC4N, MX2, PGLYRP1, MYD88 |
| negative regulation of viral genome replication | 4 | 6.35E-05 | IFITM3, IFITM2, SLPI, MX2 |
| defense response to Gram-positive bacterium | 4 | 1.50E-03 | GBP2, PGLYRP1, MYD88, GBP3 |
| immune response | 5 | 4.24E-03 | SLPI, OAS2, CMA1, PRG2, MYD88 |
| regulation of neutrophil migration | 2 | 4.86E-03 | RAC2, MYD88 |
| defense response to virus | 4 | 7.76E-03 | IFITM3, IFITM2, OAS2, MX2 |
| response to peptidoglycan | 2 | 1.93E-02 | IRAK3, MYD88 |
| response to interferon-beta | 2 | 2.17E-02 | IFITM3, IFITM2 |
| **Downregulated GO** |  |  |  |
| palate development | 3 | 2.14E-02 | MEF2C, IFT172, MSC |
| positive regulation of insulin receptor signaling pathway | 2 | 3.40E-02 | FAM132A, PRKCZ |
| cardiac muscle hypertrophy in response to stress | 2 | 4.67E-02 | MEF2C, CAMTA2 |
| vasoconstriction | 2 | 4.93E-02 | EDNRA, HTR1D |
| negative regulation of cAMP biosynthetic process | 2 | 4.93E-02 | EDNRA, OPRL1 |
| regulation of ion transmembrane transport | 3 | 4.99E-02 | KCNC4, KCNIP4, TPCN1 |
| **Upregulated KEGG** |  |  |  |
| Leukocyte transendothelial migration | 4 | 5.63E-03 | NCF4, RAC2, MMP9, PIK3CG |
| Measles | 4 | 8.34E-03 | OAS2, MX2, MYD88, PIK3CG |
| Influenza A | 4 | 1.55E-02 | OAS2, MX2, MYD88, PIK3CG |
| Leishmaniasis | 3 | 1.67E-02 | FCGR4, NCF4, MYD88 |
| Natural killer cell mediated cytotoxicity | 3 | 4.19E-02 | FCGR4, RAC2, PIK3CG |
| **Downregulated KEGG** |  |  |  |
| Neuroactive ligand-receptor interaction | 4 | 3.60E-02 | CHRM2, EDNRA, HTR1D, OPRL1 |
| Sphingolipid signaling pathway | 3 | 4.05E-02 | PRKCZ, SGPP2, MAPK12 |
