# Supplementary material for: Discovery of therapeutic targets for spinal cord injury based on molecular mechanisms of axon regeneration after conditioning lesion
Source: J Transl Med. 2023 Jul 28;21:511. doi: 10.1186/s12967-023-04375-1 (PMC10385911; doi:10.1186/s12967-023-04375-1)
Supplement: Supplementary file 5 — Additional file 5: Table S5. GO and KEGG analysis of DEGs in the young only group. [file 12967_2023_4375_MOESM5_ESM.docx]

**Table S5. GO and KEGG analysis of DEGs in the young only group**

| **Term** | **Count** | **P-Value** | **Genes** |
| --- | --- | --- | --- |
| **Upregulated GO** |  |  |  |
| DNA methylation on cytosine | 5 | 1.05E-04 | HIST2H3B, HIST1H4K, HIST1H4N, HIST2H3C1, HIST1H4F |
| DNA replication-dependent nucleosome assembly | 5 | 1.18E-04 | HIST2H3B, HIST1H4K, HIST1H4N, HIST2H3C1, HIST1H4F |
| positive regulation of gene expression, epigenetic | 5 | 1.18E-04 | HIST2H3B, HIST1H4K, HIST1H4N, HIST2H3C1, HIST1H4F |
| positive regulation of fat cell differentiation | 5 | 5.57E-04 | CEBPB, SFRP1, FNDC3B, IGF1, ZBTB7C |
| positive regulation of angiogenesis | 6 | 2.51E-03 | ECM1, LRG1, ANXA3, HSPB1, CYP1B1, CELA1 |
| nervous system development | 9 | 9.14E-03 | GPSM1, GAP43, ZEB1, ZIC1, CRMP1, SEMA4F, TCF4, IGF1, ELAVL3 |
| tissue regeneration | 3 | 1.24E-02 | GAP43, MUSTN1, APOD |
| positive regulation of apoptotic process | 8 | 1.56E-02 | ITGB1, DFFA, SFRP1, TSC22D1, ZMAT3, MMP2, CYP1B1, PHLDA3 |
| positive regulation of transcription from RNA polymerase II promoter | 15 | 2.22E-02 | CEBPB, CITED2, LUM, ZBTB38, SLC11A1, CELA1, ARRB1, IGF1, MAPK14, BACH1, PER1, ZEB1, ZIC1, TCF4, ZBTB7C |
| negative regulation of transcription from RNA polymerase II promoter | 12 | 2.63E-02 | PER1, HIST2H3B, ZEB1, CITED2, OTUD7B, TCF4, HIST2H3C1, CELA1, AEBP1, EZR, BACH1, SAP30 |
| **Downregulated GO** |  |  |  |
| regulation of ion transmembrane transport | 9 | 1.69E-05 | KCNV1, KCNB1, KCNIP2, KCNA2, CACNA2D3, KCNAB1, KCNAB2, HCN2, SCN2B |
| transmembrane transport | 11 | 9.34E-04 | KCNV1, SLC45A3, SV2B, TRPC3, SV2A, KCNB1, SLC22A17, KCNA2, TAP2, KCNAB1, HCN2 |
| brain development | 8 | 2.19E-03 | ZFHX3, NOG, ID4, KCNAB1, NRG1, TSPAN2, SMARCA1, EML1 |
| neuron fate commitment | 4 | 2.33E-03 | ISL2, ZFP521, NRG1, RUNX1 |
| neurotransmitter transport | 4 | 6.15E-03 | RIMS2, SV2B, SV2A, CPLX2 |
| negative regulation of fat cell differentiation | 4 | 7.37E-03 | ID4, C1QL4, TRIB2, ZFPM1 |
| synapse assembly | 4 | 6.15E-03 | KIRREL3, ACHE, CDH1, NRG1 |
| synaptic vesicle exocytosis | 3 | 1.07E-02 | RAB3A, SV2B, CPLX2 |
| central nervous system development | 4 | 3.79E-02 | TLX3, NOG, HAPLN1, RUNX1 |
| neurotransmitter uptake | 2 | 4.08E-02 | SV2B, SV2A |
| **Upregulated KEGG** |  |  |  |
| Systemic lupus erythematosus | 8 | 1.49E-04 | HIST1H2BN, HIST2H3B, HIST1H4K, ACTN2, HIST1H4N, HIST2H3C1, HIST2H2AA1, HIST1H4F |
| Alcoholism | 8 | 1.02E-03 | HIST1H2BN, GNG3, HIST2H3B, HIST1H4K, HIST1H4N, HIST2H3C1, HIST2H2AA1, HIST1H4F |
| Proteoglycans in cancer | 8 | 1.05E-03 | COL1A1, ITGB1, CTTN, LUM, MMP2, IGF1, MAPK14, EZR |
| Viral carcinogenesis | 7 | 9.63E-03 | HIST1H2BN, HIST1H4K, ACTN2, HIST1H4N, IL6ST, CCR5, HIST1H4F |
| Transcriptional misregulation in cancer | 6 | 9.67E-03 | CEBPB, HIST2H3B, HPGD, HIST2H3C1, IGF1, CD14 |
| Amoebiasis | 5 | 1.36E-02 | COL1A1, ACTN2, ARG1, HSPB1, CD14 |
| Leukocyte transendothelial migration | 5 | 1.40E-02 | ITGB1, ACTN2, MMP2, MAPK14, EZR |
| MicroRNAs in cancer | 7 | 2.15E-02 | MARCKS, CYP24A1, ZEB1, CYP1B1, IRS2, VIM, EZR |
| Morphine addiction | 4 | 3.72E-02 | GNG3, ARRB1, PDE7A, GABRG1 |
| Toll-like receptor signaling pathway | 4 | 4.57E-02 | SPP1, LBP, CD14, MAPK14 |
| **Downregulated KEGG** |  |  |  |
| Vascular smooth muscle contraction | 5 | 1.60E-02 | PTGIR, PRKCE, PRKCD, PRKCA, PRKG1 |
| Insulin secretion | 4 | 2.80E-02 | RIMS2, RAB3A, KCNN1, PRKCA |
| Glycerophospholipid metabolism | 4 | 3.51E-02 | ACHE, DGKZ, DGKH, CDS2 |
| Phosphatidylinositol signaling system | 4 | 3.80E-02 | PRKCA, DGKZ, DGKH, CDS2 |
