# Supplementary material for: Discovery of therapeutic targets for spinal cord injury based on molecular mechanisms of axon regeneration after conditioning lesion
Source: J Transl Med. 2023 Jul 28;21:511. doi: 10.1186/s12967-023-04375-1 (PMC10385911; doi:10.1186/s12967-023-04375-1)
Supplement: Supplementary file 6 — Additional file 6: Table S6. Predicted drugs of hub genes by DrugBank. [file 12967_2023_4375_MOESM6_ESM.docx]

**Table S6. Predicted drugs of hub genes by DrugBank**

| **Gene** | **Drug** | **Sources** | **PMIDs** |
| --- | --- | --- | --- |
| C3 | ZINC CHLORIDE | DrugBank | 23896426\|25393287 |
| C3 | METHYLDOPA | DrugBank | 23896426 |
| C3 | COPPER | DrugBank | 23896426 |
| C3 | CLOZAPINE | PharmGKB | 26503818 |
| ANXA1 | AMCINONIDE | DrugBank\|TEND | 7981624\|17139284\|17016423 |
| ANXA1 | DEXAMETHASONE | DrugBank\|NCI\|TEND | 15022342\|17644190\|17076770\|16883066\|17158208 |
| ANXA1 | HYDROCORTISONE | DrugBank\|NCI | 11142771\|1712816\|8060156\|17139284\|17016423 |
| ANXA1 | METHYLPREDNISOLONE | DrugBank\|TEND | 8060156 |
| ANXA1 | BUDESONIDE | DrugBank | 8060156 |
| ANXA1 | BETAMETHASONE PHOSPHORIC ACID | DrugBank | 8060156 |
| ANXA1 | PREDNISOLONE | TEND |  |
| ANXA1 | TRASTUZUMAB | CGI |  |
| ANXA1 | DESOXIMETASONE | TEND |  |
| ANXA1 | PREDNISONE | TEND |  |
| ANXA1 | HYDROCORTISONE PHOSPHORIC ACID | DrugBank | 11142771\|1712816\|8060156\|17139284\|17016423 |
| ANXA1 | BETAMETHASONE | TEND |  |
| ANXA1 | PREDNICARBATE | TEND |  |
| ANXA1 | TRIAMCINOLONE | TEND |  |
| ANXA1 | RIMEXOLONE | TEND |  |
| ANXA1 | HYDROCORTAMATE | TEND |  |
| ANXA1 | DESONIDE | TEND |  |
| AGTR1 | IRBESARTAN | DrugBank\|TdgClinicalTrial\|ChemblInteractions\|TEND\|GuideToPharmacology\|TTD | 7843749\|10373224\|10822210\|15101793\|14716205\|18627212\|11752352\|10069682\|10075381\|11486244\|15030294\|17408613\|10082498 |
| AGTR1 | EPROSARTAN | DrugBank\|TdgClinicalTrial\|TEND\|GuideToPharmacology\|TTD | 12782193\|12540520\|12517247\|12766389\|18093407\|11752352\|12927226\|12184062\|11320369\|10856732\|9602957 |
| AGTR1 | AZILSARTAN KAMEDOXOMIL | ChemblInteractions |  |
| AGTR1 | TELMISARTAN | DrugBank\|TdgClinicalTrial\|ChemblInteractions\|TEND\|GuideToPharmacology\|TTD | 9878991\|11408526\|15617852\|19147680\|11558835\|16938288\|15498586\|10067800\|11444497\|20448797\|9259062\|11752352\|17691961\|18580862 |
| AGTR1 | NITRENDIPINE | PharmGKB | 8952600 |
| AGTR1 | EPROSARTAN MESYLATE | ChemblInteractions |  |
| AGTR1 | CYCLOSPORINE | NCI | 17477024 |
| AGTR1 | VALSARTAN | DrugBank\|TdgClinicalTrial\|ChemblInteractions\|TEND\|GuideToPharmacology\|TTD | 8242249\|12460705\|8577935\|15579516\|12023686\|11752352 |
| AGTR1 | SARALASIN ACETATE | ChemblInteractions |  |
| AGTR1 | INDOMETHACIN | NCI | 9357777 |
| AGTR1 | LOSARTAN | DTC\|DrugBank\|TdgClinicalTrial\|TEND\|GuideToPharmacology\|PharmGKB\|TTD | 12719755\|15991937\|12710529\|16509571\|15743363\|10594793\|20436376\|15026875\|11752352\|15573751 |
| AGTR1 | OLMESARTAN MEDOXOMIL | ChemblInteractions |  |
| AGTR1 | CAPTOPRIL | PharmGKB | 19286758 |
| AGTR1 | LOSARTAN POTASSIUM | ChemblInteractions |  |
| AGTR1 | PERINDOPRIL | PharmGKB | 20712529\|8952600 |
| AGTR1 | CANDESARTAN CILEXETIL | ChemblInteractions |  |
| AGTR1 | ATORVASTATIN | NCI\|PharmGKB | 11179461 |
| AGTR1 | AZILSARTAN MEDOXOMIL | TTD |  |
| AGTR1 | SARALASIN | TTD |  |
| AGTR1 | LEVODOPA | NCI | 9458818 |
| AGTR1 | DEXAMETHASONE | NCI | 16482568 |
| AGTR1 | DISULFIRAM | DTC |  |
| CSF1 | PEXIDARTINIB | CIViC | 26222558 |
