# Supplementary figures and images for: Discovery of therapeutic targets for spinal cord injury based on molecular mechanisms of axon regeneration after conditioning lesion
Source: J Transl Med. 2023 Jul 28;21:511. doi: 10.1186/s12967-023-04375-1 (PMC10385911; doi:10.1186/s12967-023-04375-1)

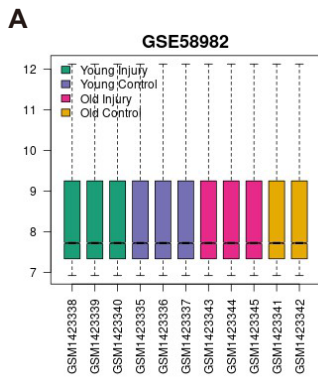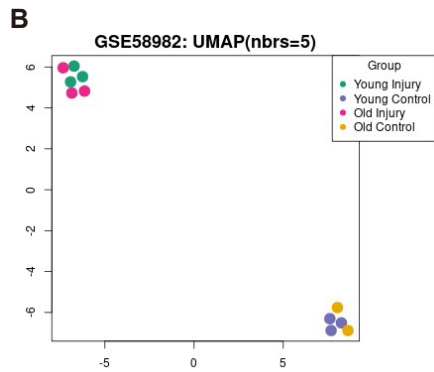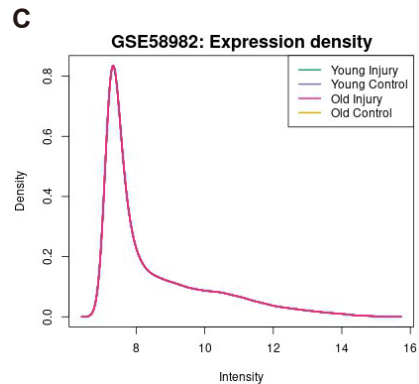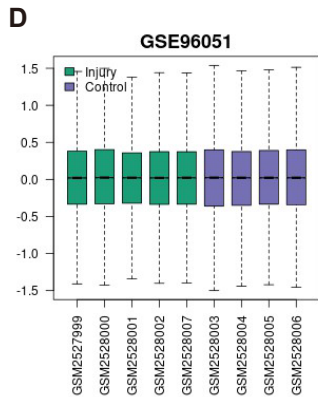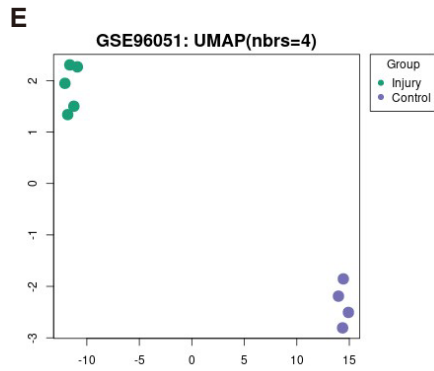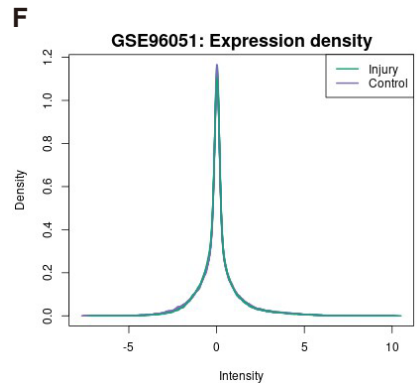

Supplement: Supplementary file 7 — Additional file 7: Figure S1. The gene expression data quality of GSE58982 and GSE96051 datasets. A The boxplots of the GSE58982 dataset. The abscissa represents the names of samples, and the ordinate represents the normalized expression levels. B The UMAP plot of the GSE58982 dataset shows the distribution relationships between the samples. C The density map of the GSE58982 dataset shows the expression of each sample. D The boxplots of the GSE96051 dataset. (E) The UMAP plot of the GSE96051 dataset. F The density map of the GSE96051 dataset. [file 12967_2023_4375_MOESM7_ESM.pdf]

A

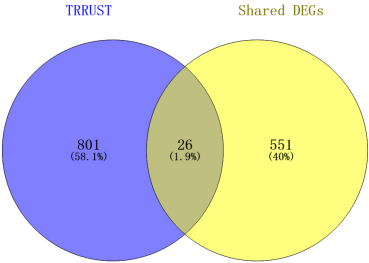

B

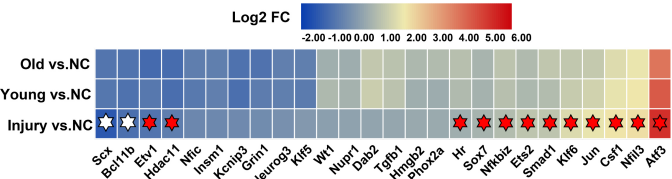

C

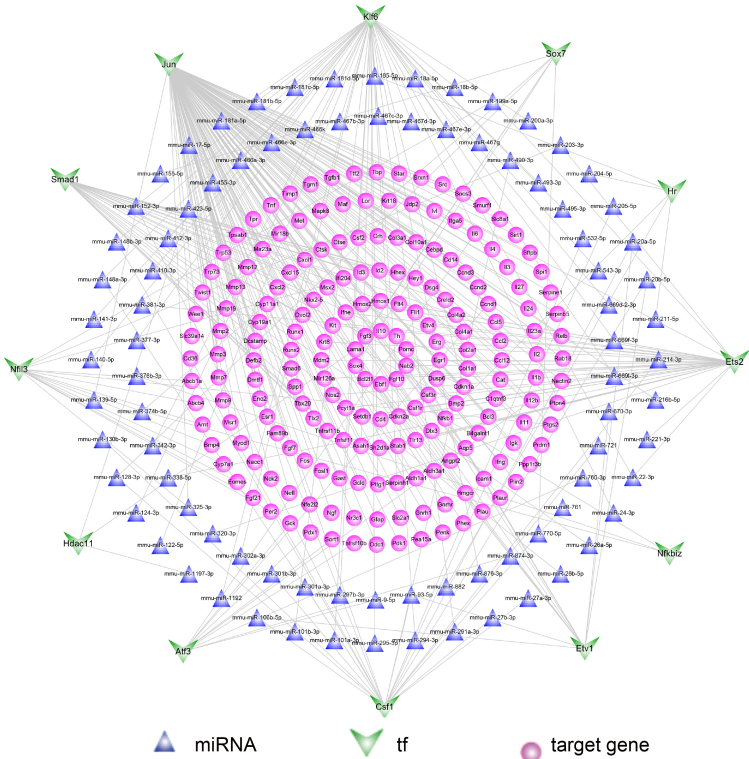

D

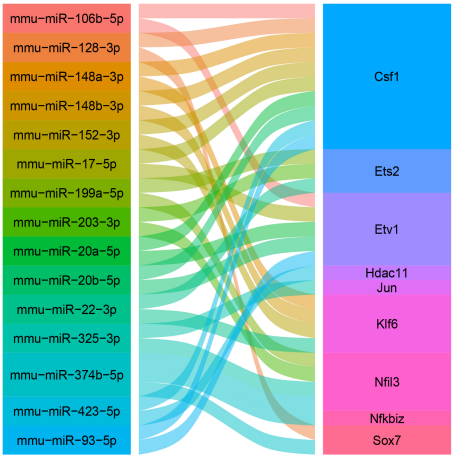

Supplement: Supplementary file 8 — Additional file 8: Figure S2. Construction of the miRNA-TF-target gene Network. A Venn diagram to obtain shared differentially expressed TFs (DEG, differentially expressed gene); B Heatmap of shared differentially expressed TFs; the differentially expressed TFs marked with asterisks were identified by the GSE96051 dataset verified, and the TFs marked with a white asterisk does not predict the targeted miRNA. C The miRNA-TF-target gene Network, blue indicates miRNAs, green indicates TFs, pink indicates mRNAs; D The hub miRNA-TF relationship pairs. (“Young vs NC”: young, 2-month-old mice, injury group vs the normal control group GSE58982; “Old vs NC”:24-month-old mice, injury group vs the normal control group in GSE58982; “Injury vs NC”: injury group vs the normal control group in GSE96051). [file 12967_2023_4375_MOESM8_ESM.pdf]
